# Steroidal Compounds at the Crossroads of Inflammation and Cancer: Implications for Drug Discovery and Therapy

**DOI:** 10.3390/biomedicines14010214

**Published:** 2026-01-19

**Authors:** Valery M. Dembitsky, Alexander O. Terent’ev

**Affiliations:** 1 Bio-Pharm Laboratories, 23615 El Toro Rd X, P.O. Box 058, Lake Forest, CA 92630, USA; 2 N.D. Zelinsky Institute of Organic Chemistry, Russian Academy of Sciences, Leninsky Prospect, 47, Moscow 119334, Russia; terentev@ioc.ac.ru

**Keywords:** anti-inflammatory, anticancer, steroids, natural products, synthetic steroids, plants, fungi, marine organisms

## Abstract

Steroidal compounds lie at the crossroads of inflammation and cancer, where modulation of common signaling pathways creates opportunities for dual-action therapeutic intervention. Accumulating evidence indicates that their anti-inflammatory and antitumor activities are frequently interconnected, reflecting shared molecular mechanisms that regulate immune signaling, oxidative stress, cell proliferation, and apoptosis. This review provides a critical and comparative analysis of major classes of bioactive steroids—including furanosteroids, neo-steroids, aromatic steroids, α,β-epoxy steroids, peroxy steroids, cyanosteroids, nitro- and epithio steroids, halogenated steroids (fluorinated, chlorinated, brominated, iodinated), and steroid phosphate esters—with emphasis on their dual anti-inflammatory and anticancer potential. More than one thousand steroidal metabolites derived from plants, fungi, marine organisms, bacteria, and synthetic sources are surveyed. While the majority exhibit either anti-inflammatory or antineoplastic activity alone, only a limited subset displays potent activity in both domains. Comparative evaluation highlights the structural features that favor dual functionality, including epoxide, peroxide, nitrile, nitro, halogen, and phosphate ester moieties, as well as rearranged or heteroatom-enriched steroidal frameworks. Where available, biological data from in vitro and in vivo assays (IC_50_ values, enzyme inhibition, cytokine modulation, and antiproliferative effects) are summarized and critically compared. Special attention is given to rare natural metabolites—such as polyhalogenated marine steroids, phosphorylated sterols, and heteroatom-containing derivatives—as well as synthetic analogues designed to enhance cytotoxic or immunomodulatory efficacy. Mechanistically, steroids exhibiting dual activity commonly modulate convergent signaling pathways, including NF-κB, JAK/STAT, MAPK, PI3K/AKT, redox homeostasis, and apoptosis regulation. Collectively, these findings underscore the potential of structurally optimized steroids as multifunctional therapeutic agents and provide a framework for the rational design of next-generation anti-inflammatory and anticancer drugs.

## 1. Introduction

Inflammation is a fundamental protective and adaptive response of the immune system in mammals, initiated by tissue injury or exposure to pathogenic agents such as bacteria and viruses. It involves coordinated cellular and molecular programs designed to eliminate harmful stimuli, restore tissue homeostasis, and promote repair [1,2,3,4,5]. As one of the earliest recognized pathological processes, inflammation was described in ancient Rome: Aulus Cornelius Celsus defined the classical cardinal signs—pain (dolor), redness (rubor), heat (calor), and swelling (tumor)—to which later authors added loss of function (amissio functionis) [6,7,8,9].

Historical medical traditions further shaped early concepts of inflammation and its management. In Ancient Greece, temple-based medicine in the *Asclepieia gradually* evolved from mysticism toward observation-driven practice under the influence of Hippocrates and his successors. Greek physicians introduced systematic diagnostics, surgical approaches, and pharmacological interventions, relying extensively on natural remedies. Dioscorides, for example, catalogued approximately 600 medicinal materials—predominantly plant-derived extracts from juices, leaves, flowers, and roots—and proposed early classification schemes based on perceived qualities such as “hot”, “cold”, “dry”, and “wet” [10,11,12,13,14,15].

In Ancient Russia, therapeutic practice similarly relied on natural products—including botanical, mineral, and animal-derived preparations—often integrated with spiritual and ritual elements. Herbal decoctions, infusions, and poultices prepared from plants such as wormwood, willow, and plantain were commonly used, accompanied by prayers, incantations, and other traditional practices; the bathhouse (banya) also served as a recognized component of health care and recovery [16,17,18].

Modern biomedical research has firmly established that inflammation is intimately linked to cancer, influencing tumor initiation, promotion, and progression [19,20,21,22]. Persistent inflammatory signaling can predispose tissues to malignant transformation, whereas the inflammatory tumor microenvironment supports proliferation, immune evasion, angiogenesis, and metastatic dissemination. Accordingly, targeting inflammatory pathways has become a key strategy in cancer prevention and therapy. Clinical and experimental studies indicate that anti-inflammatory interventions can remodel the tumor microenvironment, reduce cancer cell migration, promote apoptosis, and improve responses to chemotherapy and other anticancer modalities [23,24,25,26,27].

Against this background, steroids are of particular interest because many members of this class modulate both inflammatory signaling and cancer-related pathways. This review examines the convergence of anti-inflammatory and anticancer activities of steroidal natural products and their heteroatom-containing derivatives, emphasizing mechanistic overlap, structure–activity determinants, and the rationale for developing dual-action steroid-based therapeutics.

## 2. Furanosteroids and Their Biological Activity

Furanosteroids represent a distinct class of pentacyclic isoprenoid lipids defined by the presence of an additional furan ring fused to the steroidal core [28]. These metabolites are widely distributed among fungi, higher plants, and marine organisms, including sponges, corals, mollusks, and other invertebrates. Interest in furanosteroids has grown substantially because of their broad spectrum of biological activities, which include antibacterial, antiviral, antifungal, anti-inflammatory, and anticancer effects. Well-known representatives such as wortmannin and viridin act as potent inhibitors of phosphatidylinositol 3-kinase (PI3K), a central regulator of intracellular signaling pathways involved in cell survival, proliferation, inflammation, and oncogenesis [28,29,30,31].

A recent comprehensive survey summarized approximately 300 furanosteroids isolated from endophytic and free-living fungi, terrestrial plants, and diverse marine sources [28]. This analysis highlighted the remarkable functional diversity of this class. Many furanosteroids display pronounced cytotoxic and antiproliferative activities across a range of cancer cell lines, whereas others exhibit antiviral, antifungal, antitubercular, antibacterial, or antihepatitis properties. Importantly, several members of this group act as inhibitors of protein kinase C and phospholipase A_2_, enzymes that play key roles in inflammatory signaling and tumor progression. Together, these findings underscore the therapeutic relevance of furanosteroids and their value as structurally diverse natural scaffolds for drug discovery [28].

**Figure 1:**
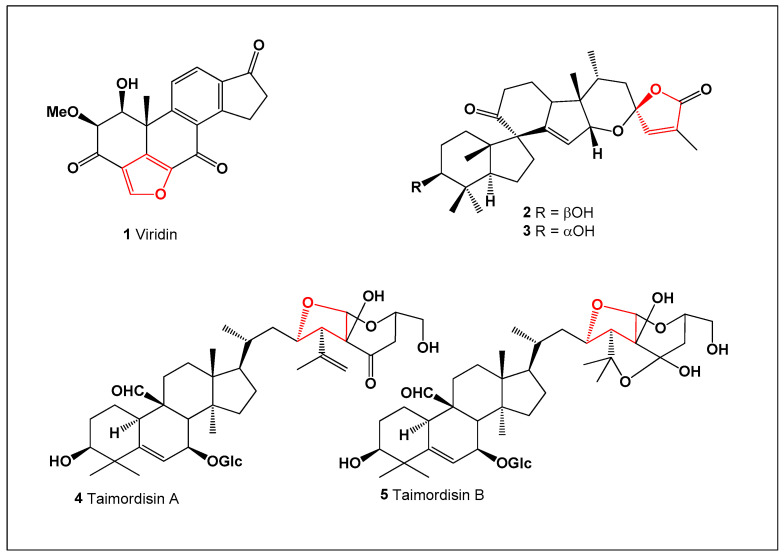
Five representative furanosteroids isolated from fungi and plants that display a pronounced predominance of anti-inflammatory activity compared with their antineoplastic effects. The characteristic furan ring, as well as its reduced analogues within the steroidal framework, are highlighted in red to emphasize this defining structural feature and its potential contribution to biological activity.

Viridin (**1**; Figure 1) is a highly fungistatic secondary metabolite produced by specific strains of *Trichoderma viride*. All viridin-producing strains examined to date synthesize this characteristic yellow pigment in significant amounts under cultivation conditions [32,33]. Although viridin shows little antibacterial activity, it exhibits strong antifungal effects, particularly against *Botrytis allii*, and its synthetic analogues retain comparable potency [34]. Beyond its antifungal properties, viridin also demonstrates marked cytotoxic and antiproliferative activity, inhibiting the growth of HUVEC, K-562, and HeLa cancer cell lines [35]. These combined biological effects highlight viridin as a representative example of the dual anti-inflammatory and antineoplastic potential found within the furanosteroid class.

Shaanxi fir (*Abies chensiensis*), native to the Chinese provinces of Gansu, Hubei, Sichuan, Tibet, and Yunnan, as well as to Arunachal Pradesh in India, produces two structurally unique abeo-steroids, spirochensilides A (**2**) and B (**3**). This rare conifer, which is cultivated outside its native range primarily at the Arnold Arboretum (USA), has attracted scientific interest because of its chemically unusual secondary metabolites. Spirochensilides A and B are the first triterpenoids reported to possess an unprecedented 8,10-cyclo-9,10-seco framework combined with extensive methyl rearrangement, thereby significantly expanding the structural diversity of naturally occurring *abeo*-steroids [36].

*Momordica charantia* (Taiwanese bitter melon), locally known as Shan Ku Gua, is a traditional vegetable and medicinal plant widely used in Taiwan and other parts of Asia, particularly for the management of diabetes. Phytochemical investigation of its fresh fruits led to the isolation of two triterpene glycosides, taimordisins A (**4**) and B (**5**), both of which exhibited strong anti-inflammatory activity in biological assays [37]. These results further emphasize the chemical richness and pharmacological relevance of this culturally important species.

**Figure 2:**
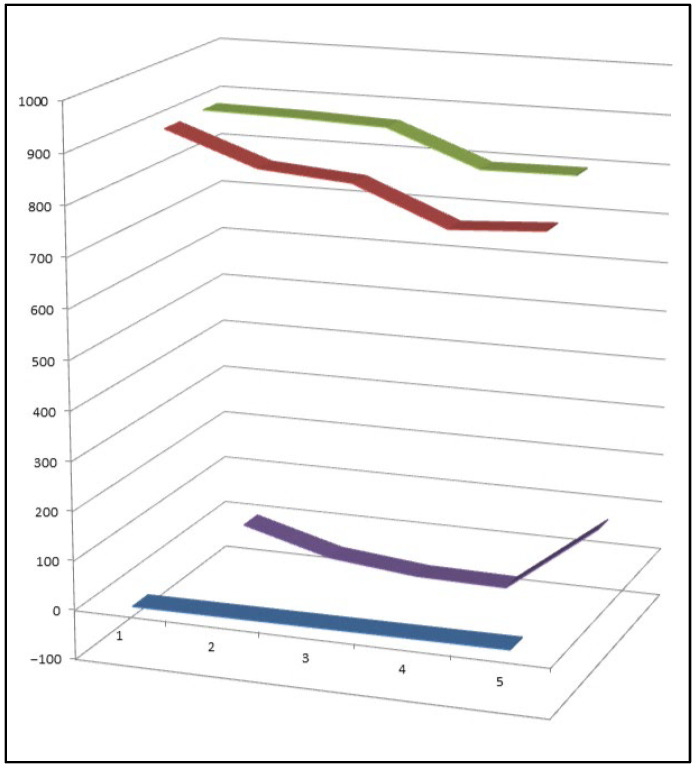
Three-dimensional activity profile of furanosteroids (**1**–**5**). The plot compares predicted anti-inflammatory (green) and antineoplastic (red) activities for each compound. Blue bars denote individual steroid numbers, while violet bars represent the calculated difference between anticancer and anti-inflammatory activities. Activity values of 800–900 indicate strong biological activity, 700–800 indicate moderate activity, and values below 700 correspond to low activity. This visualization highlights both the relative potency and the selectivity of individual furanosteroids within the series.

A comparative evaluation of approximately 300 reported furanosteroids revealed that only five compounds exhibit a marked predominance of anti-inflammatory activity over antineoplastic effects (Figure 2). This observation highlights the relative rarity of furanosteroids with selective anti-inflammatory profiles and underscores their particular interest as leads for inflammation-targeted drug development [28].

## 3. Neo-Steroids and Their Biological Activity

Secondary metabolites containing a *tert*-butyl group (or *tert*-butyl fragment) are relatively rare in nature. Although this structural motif has been reported in certain cyanobacteria, it is also present in a limited number of plant, fungal, algal, and marine invertebrate metabolites [38,39,40,41]. Owing to its pronounced steric bulk, the *tert*-butyl group is widely used in synthetic organic chemistry to confer kinetic stabilization. Its strong steric and electronic effects—often referred to as the *tert*-butyl effect—can significantly influence molecular conformation, reactivity, and interactions with biological targets [42,43,44,45].

**Figure 3:**
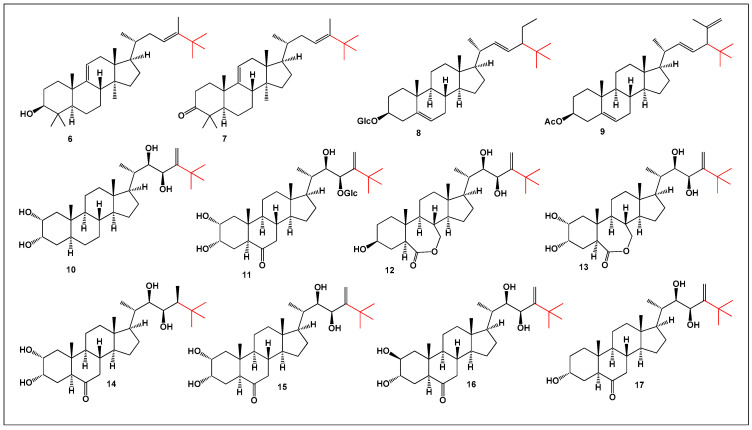
Analysis of the published literature covering more than 100 reported neo-steroids revealed that only twelve compounds (**6**–**17**) exhibit consistently strong biological activity. These selected neo-steroids demonstrate predominantly pronounced antineoplastic effects accompanied by significant anti-inflammatory activity, distinguishing them from the majority of structurally related metabolites with weaker or more selective biological profiles. The tert-group of steroids is highlighted in red.

Neo-steroids (Figure 3) represent a distinctive class of natural or synthetic lipids characterized by the incorporation of a *tert*-butyl substituent into the steroidal carbon framework [46]. These structurally uncommon steroids have been isolated from diverse biological sources, including higher plants, fungi, and marine sponges. Increasing evidence indicates that *neo*-steroids exhibit a broad spectrum of biological activities, with reported anticancer, neuroprotective, and hepatoprotective effects, highlighting their potential pharmacological relevance [46,47,48].

**Figure 4:**
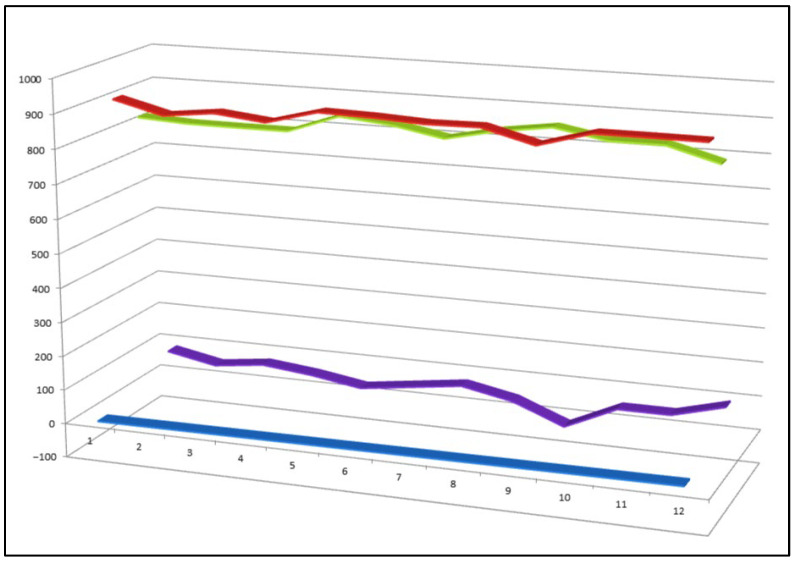
Three-dimensional activity profile of twelve *neo*-steroids (**6**–**17**), illustrating the predominance of anticancer activity (red) over anti-inflammatory activity (green). Although both biological effects fall within the strong activity range, quantitative comparison reveals that anticancer activity exceeds anti-inflammatory activity by approximately 5.1%. This close yet consistent difference suggests that *tert*-butyl-substituted steroid frameworks preferentially enhance antiproliferative mechanisms while largely retaining anti-inflammatory potential, supporting their relevance as dual-action bioactive scaffolds. In this and all subsequent figures, blue bars denote individual steroid numbers, while violet bars represent the calculated difference between anticancer and anti-inflammatory activities.

Species of the genus *Quercus* are a rich source of secondary metabolites and provide abundant biomaterials used in pharmaceutical, cosmetic, and food-related applications. Chemical investigation of the stems of *Quercus bambusaefolia*, *Q. championi*, and *Q. myrsinaefolia* led to the isolation of two neo-steroidal triterpenoids: 24,25-dimethyl-9(11),23-lanostadienol (**6**; structure shown in Figure 3, activity in Figure 4) and 24,25-dimethyl-lanosta-9(11),23-dien-3-one (**7**) [49]. These compounds further expand the structural diversity of naturally occurring *tert*-butyl-containing steroids and underscore the chemical richness of the *Quercus* genus.

Glucoside (**8**) was identified within the triterpene alcohol fraction of the unsaponifiable lipid components obtained from 28 different plant materials, encompassing seeds, leaves, stems, fruit pericarps, roots, and mature tissues across 13 genera of the family *Cucurbitaceae* [50]. The widespread occurrence of this glucosylated neo-steroid suggests that *tert*-butyl-containing triterpenoids may play conserved physiological or defensive roles in cucurbit plants and highlights their importance as chemotaxonomic markers within this family.

In the marine environment, the sponge *Halichondria* sp., collected from the Sea of Japan, was found to produce an unusual neo-steroidal metabolite, (3β,22E,24ξ)-28,28-dimethyl-stigmasta-5,22,25-trien-3-ol acetate (**9**). Organic extracts of this sponge exhibited both anti-inflammatory and antineoplastic activities, underscoring marine sponges as prolific sources of sterically congested and biologically active lipids that are rarely encountered in terrestrial organisms [51].

Brassinosteroids represent a well-characterized class of plant phytohormones that regulate a wide range of physiological and developmental processes, including cell elongation, rhizogenesis, seed germination, flowering, and senescence. In addition to their growth-promoting roles, brassinosteroids significantly enhance plant tolerance to abiotic stresses such as salinity, drought, and temperature extremes [52,53,54,55]. Several neo-steroids (**10**–**17**) belonging to this structural group have been isolated from the seeds of *Phaseolus vulgaris*, further illustrating the chemical diversity and broad distribution of *tert*-butyl-containing steroids in the plant kingdom and supporting their relevance not only in plant biology but also as potential leads for pharmacological exploration [56,57,58].

## 4. Aromatic Steroids and Their Biological Activity

Aromatic steroids represent a structurally diverse class of natural and synthetic lipids defined by the incorporation of one or more aromatic rings into the steroidal framework. This aromatization profoundly alters the electronic distribution and conformational rigidity of the molecule, often resulting in modified receptor interactions and distinct biological profiles. Aromatic steroids are widely distributed across multiple biological kingdoms—including plants, animals, fungi, and bacteria—and have also been detected in geological matrices such as marine sediments and petroleum deposits, where they serve as valuable chemotaxonomic and geochemical biomarkers [59,60,61,62].

From a pharmacological perspective, aromatic steroids are of particular interest due to their broad spectrum of biological activities, which include antitumor, anti-inflammatory, antimicrobial, antiviral, and neuroprotective effects. The presence of an aromatic ring within the steroid nucleus can enhance π–π stacking interactions, alter redox behavior, and improve binding affinity to enzymes and nuclear receptors involved in inflammation and cancer-related signaling pathways. Consequently, aromatic steroids often display activity profiles that differ markedly from those of fully aliphatic sterols.

**Figure 5:**
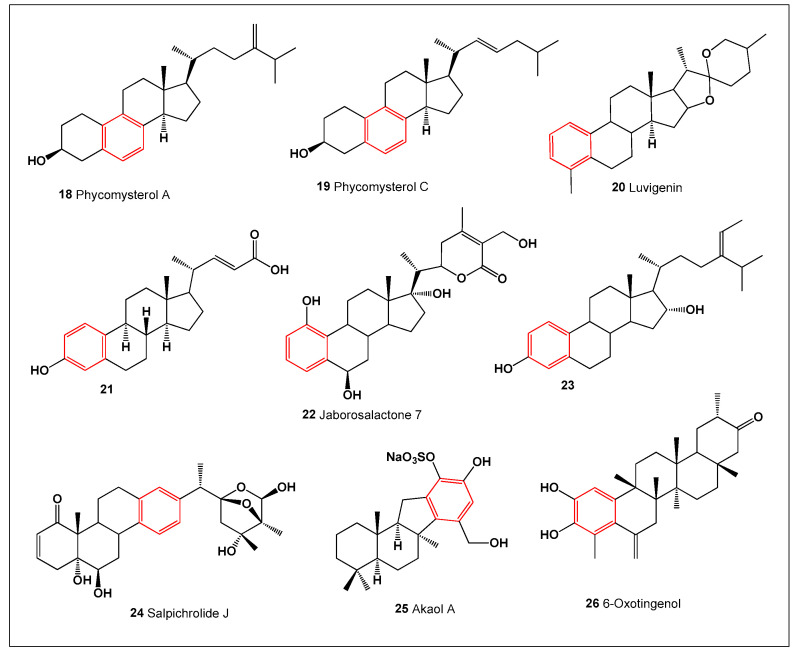
Summary of biological activity trends among aromatic steroids. From the analysis of more than 90 reported aromatic steroids, only nine compounds (**18**–**26**) were identified as exhibiting predominantly strong antitumor activity. In contrast, steroids **20**, **22**, **24**, and **26** showed a clear predominance of anti-inflammatory activity over antineoplastic effects. This distribution highlights the functional divergence within aromatic steroid scaffolds and underscores how subtle structural variations can shift biological selectivity between inflammation-related and cancer-related targets. The tert-group of steroids is highlighted in red. The comparative activity profiles of these aromatic steroids are illustrated in Figure 6.

**Figure 6:**
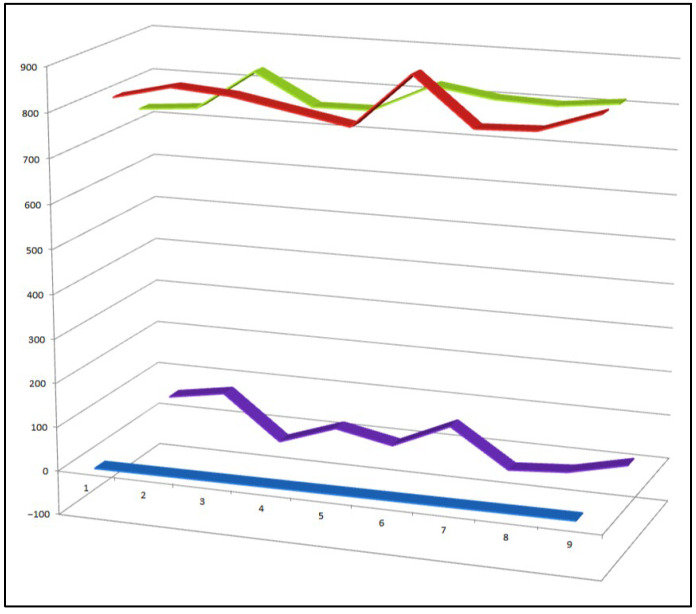
Three-dimensional activity profile of nine aromatic steroids (**18**–**26**), illustrating the relationship between anticancer and anti-inflammatory activities. Red bars represent antineoplastic activity, while green bars indicate anti-inflammatory activity for each compound. The plot demonstrates a clear positive correlation between the two bioactivities across this subset of aromatic steroids, with most compounds exhibiting values in the potent to moderate activity range. This pattern suggests that incorporation of aromatic ring systems into the steroidal framework may favor dual modulation of inflammatory and tumor-related pathways, supporting the concept of shared structure–activity determinants for these biological effects.

The filamentous fungus *Phycomyces blakesleeanus* (order *Mucorales*, phylum *Zygomycota*) is a well-documented producer of monoaromatic steroids. Among these metabolites, phycomysterols A (**18**) and C (**19**) (structures shown in Figure 5; biological activity summarized in Figure 6) are particularly noteworthy. Both compounds possess a rare 19-norergostane skeleton featuring an aromatic B-ring, a structural motif seldom encountered in natural steroids. Phycomysterol A has demonstrated promising anti-HIV activity, highlighting the therapeutic potential of fungal-derived aromatic steroids and reinforcing the importance of fungi as sources of structurally and biologically unique steroidal metabolites [63].

A spirostan-type sapogenin, luvigenin (**20**), represents a further example of a naturally occurring aromatic steroid with a broad phylogenetic distribution. This compound has been isolated from several botanically unrelated taxa, including the monotypic genus *Metanarthecium luteoviride* (family Nartheciaceae) [64], the perennial evergreen *Yucca gloriosa* [65], and the ornamental onion *Allium giganteum* [66]. Its occurrence across distinct plant lineages suggests the operation of conserved, albeit uncommon, biosynthetic routes capable of generating aromatic modifications within the steroidal framework. Such convergence implies an underlying evolutionary advantage, possibly related to defense or signaling functions.

Additional aromatic and partially aromatic steroids have been identified from a wide range of terrestrial and marine organisms, further underscoring their structural diversity and biological relevance. A bile acid-derived aromatic steroid, 3-hydroxy-19-norchola-1,3,5(10),22-tetraen-24-oic acid (**21**), was isolated from aqueous–ethanolic extracts of the marine sponge *Sollasella moretonensis*, collected off the coast of northern Queensland, Australia [67]. This finding highlights marine sponges as important sources of metabolically modified steroidal structures.

Among plant-derived aromatic steroids, a bioactive withanolide, jaborosalactone 7 (**22**), was isolated from the roots of *Jaborosa magellanica* (Solanaceae) [68], while a closely related withanolide, salpichrolide J (**24**), was detected in extracts of *Salpichroa origanifolia* [69]. Withanolides are well recognized for their cytotoxic and anti-inflammatory properties, and the presence of aromatic features within these molecules may contribute to their interaction with key cellular targets.

The bark of the tropical tree *Terminalia catappa* (Combretaceae) yielded an unusual aromatic steroid, compound **23**, and leaf extracts of this species demonstrated notable antimicrobial activity against *Escherichia coli*, *Staphylococcus aureus*, and *Candida albicans*, as well as antifungal effects against *Epidermophyton floccosum* and *C. albicans* [70]. These activities support the ecological role of aromatic steroids in plant defense mechanisms.

From marine sources, a sesquiterpene quinol, compound **25**, was obtained from a sponge of the genus *Aka* collected in the Federated States of Micronesia [71]. Finally, extracts of the leaves and roots of *Maytenus ilicifolia* (“Espinheira Santa”), a plant widely used in Brazilian traditional medicine, exhibited pronounced anticancer activity and led to the isolation of the aromatic steroid 6-oxotingenol (**26**) [72]. Collectively, these examples illustrate how aromatic steroids span diverse biosynthetic origins while consistently contributing to anti-inflammatory, antimicrobial, and anticancer activity profiles.

## 5. α,β-Epoxy Steroids and Their Biological Activity

The α,β-epoxy (oxirane) group is a prominent functional motif in organic chemistry, defined by an oxygen atom bridging two adjacent carbon atoms to form a highly strained three-membered ring [73,74,75]. This intrinsic ring strain, combined with the electron-rich nature of the oxygen atom, renders epoxides highly reactive toward nucleophilic attack, ring-opening processes, rearrangements, and stereoselective transformations [76,77,78]. When incorporated into a steroidal framework, the epoxide moiety can profoundly influence molecular conformation, electronic distribution, and reactivity, often translating into enhanced or altered biological activity.

Steroids bearing an α,β-epoxide function constitute a structurally diverse group of natural and synthetic compounds isolated from fungi, plants, animals, marine invertebrates, and microorganisms [59,62]. Introduction of the epoxide ring modifies the physicochemical properties of the steroid nucleus and facilitates interactions with a broad range of biological targets, including enzymes, receptors, transcription factors, and intracellular signaling proteins. As a result, α,β-epoxysteroids frequently display cytotoxic, anti-inflammatory, antiviral, and enzyme-inhibitory activities, and in some cases exhibit multitarget pharmacological profiles [79,80,81,82].

**Figure 7:**
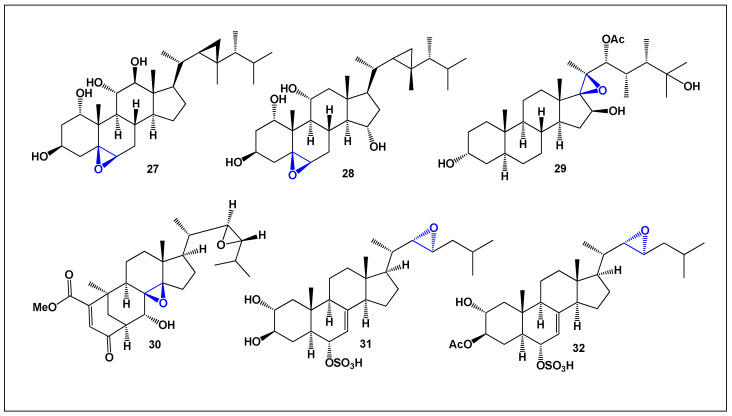
Among more than 200 reported steroids containing an α,β-epoxide (oxirane) moiety, only six compounds (**27**–**32**) were identified as exhibiting pronounced biological activity relevant to this review. Most of these epoxysteroids show a clear predominance of antitumor activity; however, compounds **28**, **29**, and **30** are notable exceptions, as they display stronger anti-inflammatory effects than antineoplastic ones. This distribution highlights the rarity of epoxysteroids with selective or dual bioactivity and underscores the importance of subtle structural variations in determining biological outcomes. Blue indicates the presence of α,β-Epoxy groups in Steroids. Quantitative activity comparisons for these compounds are summarized in Figure 8.

**Figure 8:**
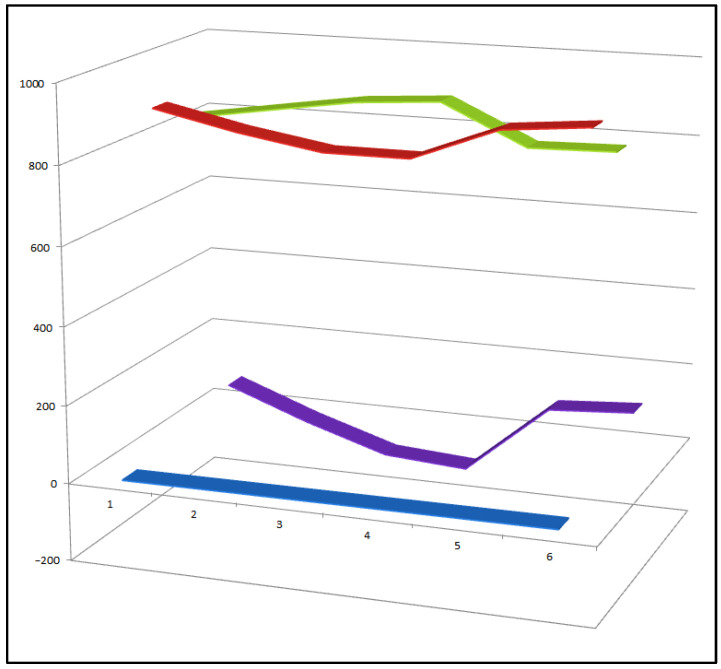
Three-dimensional activity profile of six α,β-epoxysteroids (**27**–**32**), illustrating the relationship between anticancer activity (red) and anti-inflammatory activity (green). The plot demonstrates a general correlation between the two activities, with activity values for both endpoints falling within the strong activity range (800–910). This visualization emphasizes the potential of selected epoxysteroids to act on overlapping molecular pathways involved in tumor progression and inflammation.

A systematic survey of more than 250 reported α,β-epoxysteroids revealed that only six compounds (**27**–**32**, Figure 7) combine pronounced anti-inflammatory activity with potent antineoplastic effects (Figure 8). Although numerically limited, this subset is particularly noteworthy, as it highlights specific structural features that may enable simultaneous modulation of inflammatory and cancer-related pathways, positioning these molecules as attractive candidates for further pharmacological optimization.

Two representative members of this group, the polyhydroxylated sterols **27** and **28**, were isolated from the gorgonian coral *Isis hippuris* (sea bamboo), a marine organism widely distributed in the western Pacific Ocean, especially near Indonesia. In addition to their dual anti-inflammatory and anticancer profiles, both compounds demonstrated significant antiviral activity against human cytomegalovirus (HCMV), underscoring the therapeutic versatility of marine-derived epoxy steroids [83,84].

Fungal endophytes also contribute substantially to the diversity of α,β-epoxysteroids. From the plant-associated strain *Phomopsis* sp. TJ507A (family Diaporthaceae), an unusual ergostane-type steroid named phomopsterone A (**29**) was isolated [85]. This metabolite exhibits a highly rearranged carbon skeleton, reflecting the remarkable biosynthetic plasticity of endophytic fungi. A second, structurally distinct compound originally reported under the same name has been redesignated here as phomopsterone B (**30**) to avoid confusion. This steroid features a rare rearranged bicyclo [3.3.1]nonane system formed via B-ring scission followed by a 180° rotation of the A-ring during biosynthesis [86]—a transformation that is exceedingly uncommon in natural products and highlights the chemical novelty accessible through fungal metabolism.

Marine invertebrates further expand the repertoire of α,β-epoxysteroids. Two structurally unusual sterols, hippuristeroketal A (**31**) and hipposterol G (**32**), were isolated from the Taiwanese octocoral *Isis hippuris*. Both compounds exhibited inhibitory activity against HCMV, with EC_50_ values in the range of 6–8 μg/mL, providing additional evidence that epoxy-containing marine steroids can effectively interfere with viral replication while also exhibiting anti-inflammatory and antineoplastic potential [87].

These examples demonstrate that α,β-epoxy steroids, although relatively rare among known steroidal metabolites, represent a highly promising class of bioactive compounds. Their distinctive structural features and capacity to engage multiple biological targets support continued investigation into their mechanisms of action and their development as dual-acting anti-inflammatory and anticancer agents.

## 6. Peroxy Steroids and Their Biological Activity

Peroxy steroids constitute a distinctive and chemically intriguing class of natural products defined by the presence of a peroxide linkage (R–O–O–R) embedded within a steroidal or triterpenoid framework. The peroxide functionality confers pronounced chemical reactivity, enabling redox-driven interactions with biological targets, which often translate into potent and diverse biological effects. Peroxy steroids are biosynthesized by a wide range of organisms, including terrestrial plants, fungi, algae, and marine invertebrates, and have been associated with antineoplastic, antiprotozoal (particularly antimalarial), anti-inflammatory, antitrypanosomal, and antibacterial activities [88,89,90,91].

**Figure 9:**
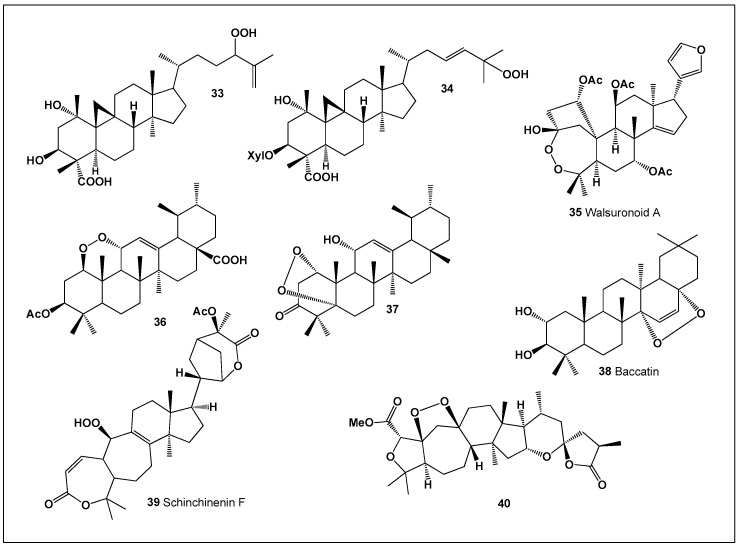
Among the approximately 400 peroxy steroids identified from natural sources to date, only eight compounds (**33**–**40**) have been found to exhibit a clear combination of both anti-inflammatory and antitumor activities. This small proportion highlights the rarity of dual-action peroxy steroids and underscores the importance of specific structural features—such as the position of the peroxide linkage and the overall oxidation pattern of the steroidal scaffold—in governing their biological selectivity and potency. Comparative activity of steroids is shown in Figure 10.

Despite the structural diversity of this class—currently encompassing approximately 400 characterized peroxy steroids—only a small subset has been shown to display both strong anti-inflammatory and antineoplastic activities. To date, just eight compounds meet this dual-activity criterion, underscoring both the rarity and the pharmacological significance of such metabolites (Figure 9) [89,92,93]. This observation suggests that precise positioning of the peroxide moiety and the overall oxidation pattern of the steroid nucleus are critical determinants of biological selectivity and potency.

**Figure 10:**
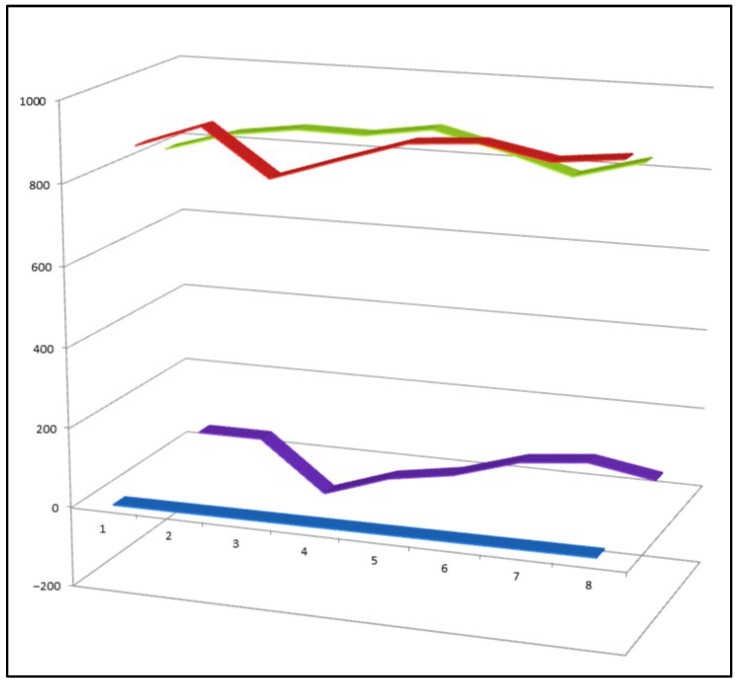
Three-dimensional activity profile of eight bioactive peroxy steroids (**33**–**40**), illustrating the relationship between anticancer (red) and anti-inflammatory (green) activities. In most cases, anticancer activity predominates, with calculated values generally exceeding those for anti-inflammatory effects. Notable exceptions are compounds **34**, **35**, and **36**, which display a more balanced or comparatively enhanced anti-inflammatory profile. For all eight compounds, both activity parameters fall within the strong biological activity range (800–920), emphasizing the pharmacological relevance of this rare subgroup of peroxy steroids.

Several representative examples illustrate the chemical and biological breadth of peroxy steroids. *Markhamia lutea* (Bignoniaceae), commonly known as the Nile tulip or siala tree and native to East Africa, produces bioactive triterpenoids such as musambin A (**33**) and its glycosylated analogue musambioside B (**34**). These compounds exhibit notable antiplasmodial and antitrypanosomal activities, supporting the traditional medicinal use of the plant and highlighting the therapeutic relevance of plant-derived peroxy triterpenoids [94].

A structurally exceptional peroxy-limonoid, walsuronoid A (**35**), was isolated from *Walsura robusta*. This metabolite possesses an unprecedented 3,4-peroxide-bridged A-seco skeleton, representing a rare biosynthetic modification within the limonoid family. Although its antimalarial activity is relatively weak, walsuronoid A significantly expands the known structural space of peroxide-containing steroids and provides insight into alternative oxidative pathways in limonoid biosynthesis [95].

Additional peroxy triterpenoids have been reported from diverse plant sources. A taraxastane-type triterpene (**36**) was isolated from the aerial roots of *Ficus microcarpa* [96], while an ursane-type peroxide, 1α,5α-dioxy-11α-hydroxyurs-12-en-3-one (37), was obtained from the rhizomes of *Vladimiria muliensis*. Compound **37** displayed modest but broad-spectrum antimicrobial activity against both Gram-positive and Gram-negative bacteria, as well as *Candida albicans*, indicating that peroxide incorporation may contribute to membrane- or redox-mediated antimicrobial mechanisms [97].

Although not itself biologically active as an anticancer agent, baccatin (**38**)—a diterpenoid isolated from yew trees (*Taxus* spp., particularly *T. baccata*)—deserves special mention as the indispensable biosynthetic precursor of paclitaxel and docetaxel. These clinically essential chemotherapeutics underscore the indirect but profound medical importance of peroxide-containing or highly oxygenated diterpenoid scaffolds [98].

Further expanding the functional diversity of peroxy steroids, schinchinenin F (**39**), a highly oxygenated triterpenoid bearing a rare hydroperoxyl group, was isolated from species of the genus *Schisandra*. This compound exhibited antiviral activity against HSV-2 and adenovirus, suggesting that hydroperoxide-bearing triterpenoids may interfere with viral replication or entry processes [99]. Finally, the triterpene peroxide pseudolarolide T1 (**40**), isolated from the leaves of *Pseudolarix kaempferi* (Pinaceae), adds to the growing inventory of plant-derived peroxide-containing triterpenoids and further illustrates the biosynthetic versatility of this class [100].

In the aggregate, these examples demonstrate that peroxy steroids, although relatively rare, occupy a privileged position in natural product chemistry. Their unique redox-active functional groups, combined with diverse steroidal scaffolds, make them particularly attractive as leads for the development of novel anti-inflammatory, anticancer, antiprotozoal, and antiviral agents.

## 7. Cyanosteroids and Their Biological Activity

Nitrile-containing metabolites are broadly distributed in nature and have been identified in algae, fungi, plants, insects, and numerous microorganisms, reflecting their diverse biosynthetic pathways and ecological functions [101,102,103]. In contrast, higher animals do not biosynthesize nitrile-containing secondary metabolites [104]. Naturally occurring nitrile-bearing compounds include cyanogenic glycosides, cyanolipids, and aromatic nitriles, many of which play defensive or signaling roles and have attracted sustained interest because of their structural diversity and pharmacological relevance [105,106,107,108].

Cyanolipids were first described in 1920 from seeds of flowering plants belonging to the order Sapindales, particularly species native to the Indian subcontinent and Southeast Asia. These metabolites originate from amino acid-derived biosynthetic pathways and are now known to be relatively widespread in the plant kingdom. In striking contrast, cyanosteroids—defined as steroids bearing one or more nitrile (–CN) groups—have not been identified as natural products. All known cyanosteroids are synthetic in origin, underscoring the rarity of nitrile incorporation into the steroidal framework in biological systems [109].

The first anabolic cyanosteroids were synthesized in the 1940s–1950s, representing an important milestone in steroid chemistry and medicinal research [109,110,111]. Introduction of the nitrile group into the steroid nucleus was shown to significantly alter electronic properties, receptor interactions, and metabolic stability. As a result, cyanosteroids rapidly became valuable tools for probing structure–activity relationships and for developing compounds with enhanced biological potency. To date, more than 460 synthetic cyanosteroids have been reported in the literature, many of which exhibit notable antibacterial, antifungal, anti-inflammatory, and anticancer activities [112,113,114,115,116].

**Figure 11:**
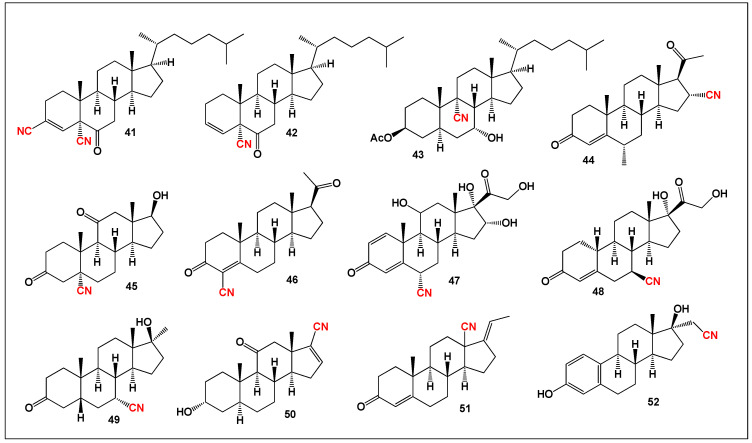
Fourteen representative synthetic cyanosteroids (**41**–**52**) selected from the literature that demonstrate pronounced anti-inflammatory and antitumor activities. These compounds exemplify how incorporation of a nitrile (–CN) group into the steroidal framework can markedly enhance biological potency and confer dual activity against inflammatory and neoplastic processes.

**Figure 12:**
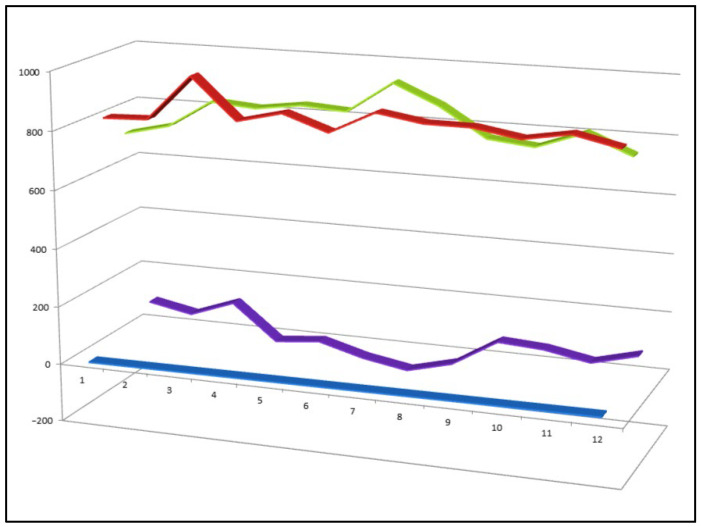
Three-dimensional activity profile of twelve bioactive cyanosteroids (**41**–**52**), illustrating the comparative balance between anticancer (red) and anti-inflammatory (green) effects. For most compounds, anticancer activity predominates, whereas cyanosteroids **44**, **47**, **48**, and **49** display relatively stronger or more balanced anti-inflammatory responses. Activity values for both biological endpoints fall within the range of 800–970, corresponding to moderate-to-strong predicted activity and underscoring the therapeutic potential of this class of synthetic steroids.

Among this large body of synthetic analogues, only a limited subset demonstrates pronounced dual anti-inflammatory and antineoplastic activity. Representative examples of such bioactive cyanosteroids (**41**–**52**) are shown in Figure 11, while their comparative biological activity profiles are summarized in Figure 12. Together, these data highlight the pharmacological relevance of nitrile substitution in steroidal scaffolds and emphasize the potential of cyanosteroids as leads for the development of multifunctional therapeutic agents targeting both inflammation and cancer.

## 8. Nitro-Steroids and Their Biological Activity

Nitro-containing organic compounds, defined by the presence of a nitro functional group (–NO_2_) covalently bound to a carbon atom, occur in both natural and synthetic contexts. Although the majority of nitro compounds encountered in chemistry and pharmacology are synthetic, a considerable number of naturally occurring nitro metabolites have been identified across diverse biological systems, including plants, fungi, and bacteria [117,118,119,120]. These natural nitro compounds often play roles in chemical defense, microbial competition, and signaling.

Among naturally occurring nitro metabolites, aristolochic acids and their derivatives from species of the genus *Aristolochia* (Aristolochiaceae) are among the most extensively studied. In addition, several *Streptomyces* and *Penicillium* species produce nitro-containing secondary metabolites that range from relatively simple nitro-aromatic compounds to structurally complex molecules such as siderophores, alkaloids, and cyclic peptides. According to ChemNetBase, more than 1000 natural nitro-containing metabolites have been catalogued to date, underscoring the chemical diversity and biological relevance of this functional group in nature [121,122,123,124,125,126].

The nitro group occurs in a wide array of molecular frameworks, including aliphatic and aromatic hydrocarbons, fatty acids, terpenoids, heterocycles, and peptides [121]. As a strongly electron-withdrawing substituent, the –NO_2_ group exerts a pronounced influence on molecular polarity, redox behavior, and reactivity. It can stabilize negative charge, participate in redox cycling, and serve as a precursor to other functional groups through reduction or rearrangement reactions. These properties make the nitro group a powerful tool in synthetic organic chemistry and an important determinant of biological activity in medicinal chemistry [127,128].

**Figure 13:**
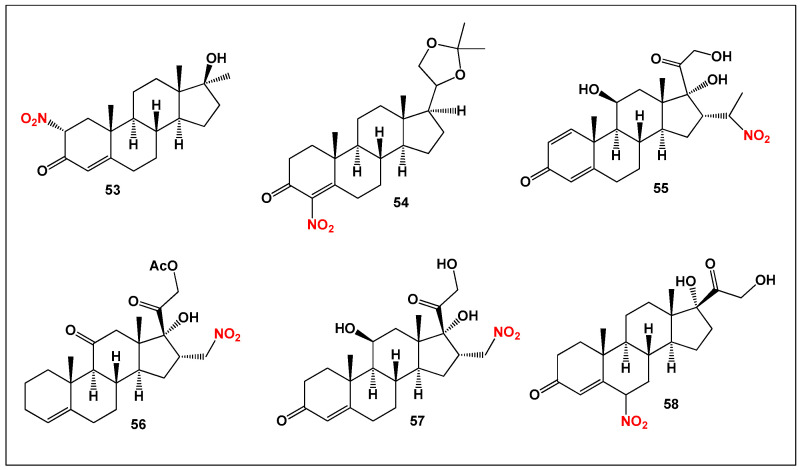
Representative synthetic nitro-steroids (**53**–**58**) that exhibit both anti-inflammatory and antitumor activities. These compounds illustrate how incorporation of a nitro (–NO_2_) group into the steroidal framework can substantially enhance biological potency. Structural variation among the nitro-steroids—particularly the position of the nitro substituent and the oxidation pattern of the steroid nucleus—plays a key role in determining the balance between anti-inflammatory and anticancer effects. Quantitative comparisons of their biological activities are summarized in Figure 14.

Nitro-steroids—steroidal molecules (Figure 13) incorporating one or more nitro substituents—do not occur naturally and are entirely synthetic in origin. They were first prepared more than six decades ago and have since attracted sustained interest due to their distinctive pharmacological profiles [127,128,129,130]. Nitro substitution within the steroid nucleus can significantly alter receptor binding, metabolic stability, and redox activity, leading to enhanced or novel biological effects. Reported activities of nitro-steroids include anti-arthritic, anti-inflammatory, antiproliferative, and anticancer effects, with several compounds demonstrating the ability to modulate inflammatory signaling pathways while simultaneously inhibiting tumor cell growth.

**Figure 14:**
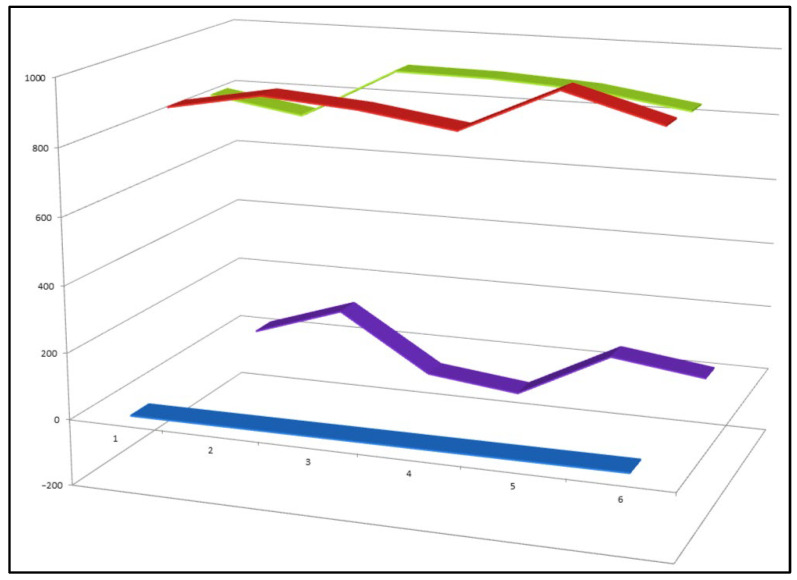
Three-dimensional activity profile of six synthetic nitro-steroids (**53**–**58**), illustrating the relationship between anticancer (red) and anti-inflammatory (green) activities. For most compounds, anticancer activity predominates, with calculated activity values ranging from 800 to 980, corresponding to strong biological activity. Steroids **55** and **56** represent notable exceptions, displaying a more balanced or relatively enhanced anti-inflammatory profile. This visualization highlights how subtle structural modifications within nitro-steroids can shift the dominance between antitumor and anti-inflammatory effects.

To date, more than 150 synthetic nitro-steroids have been reported in the literature. However, only a small subset—six compounds (**53**–**58**)—has been identified as exhibiting both pronounced antineoplastic and anti-inflammatory activity (Figure 14). This limited overlap highlights the stringent structural requirements necessary for achieving dual biological activity and emphasizes the importance of nitro group positioning, oxidation state, and overall steroidal framework in governing pharmacological outcomes.

## 9. Epithio Steroids and Their Biological Activity

**Figure 15:**
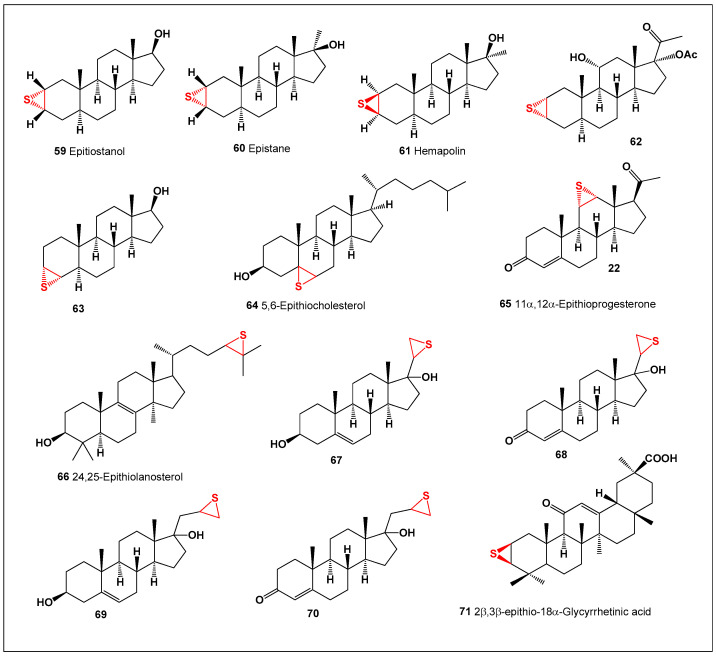
Thirteen representative synthetic epithio steroids (**59**–**71**) selected from a larger dataset of approximately fifty epithio-containing steroids with reported biological activity. Comparative analysis indicates that, within this subset, antineoplastic activity consistently predominates over anti-inflammatory effects. The quantitative activity profiles of these compounds are summarized and visualized in Figure 16.

Thiirane-containing compounds occupy an important niche in modern organic, medicinal, and bioorganic chemistry, owing to the high ring strain and electrophilic character of the three-membered sulfur-containing epithio (thiirane) moiety [131,132,133,134,135,136,137,138]. When incorporated into lipophilic frameworks such as steroids, this functionality can profoundly influence molecular reactivity, target engagement, and biological potency. Epithio steroids (compounds **59**–**70**, and the triterpenoid **71**, Figure 15) represent a rare and structurally distinctive class of semi-synthetic or fully synthetic steroidal derivatives. To date, no naturally occurring epithio steroids have been identified, and all known examples arise from targeted chemical modification of pre-existing steroidal scaffolds [136,137,138]. Their pronounced lipophilicity and limited aqueous solubility further distinguish them from most naturally occurring steroids and contribute to unique pharmacokinetic and biological profiles.

**Figure 16:**
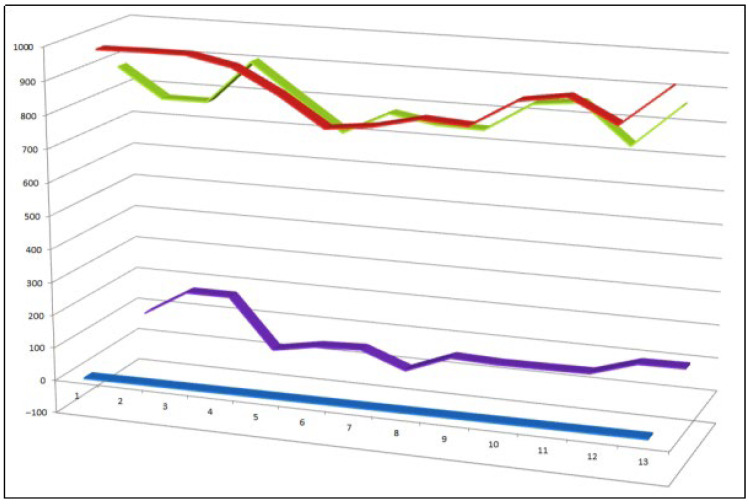
Three-dimensional activity profile of thirteen synthetic epithio steroids (**59**–**71**), illustrating the predominance of anticancer activity (red) relative to anti-inflammatory activity (green). Anticancer activity values fall largely within the strong activity range (800–970), whereas anti-inflammatory activity is generally lower and, in several cases, falls within the moderate activity range. This visualization highlights the pronounced cytotoxic bias of thiirane-modified steroidal frameworks.

Epithio steroids have attracted considerable attention due to their marked cytotoxic and antiproliferative activities, positioning them as promising candidates in anticancer drug discovery. The thiirane ring is thought to play a central role in these effects, acting as a reactive electrophilic center capable of interacting with nucleophilic residues in proteins, enzymes, or regulatory signaling molecules. In many cases, the presence of an epithio group at the C-2/C-3 position of the steroid nucleus confers enhanced biological activity and is a defining feature of several anabolic steroid analogues extensively studied in pharmacology, endocrinology, and sports medicine [138,139,140,141].

A well-studied natural precursor for the synthesis of epithio steroids is glycyrrhetinic acid, a pentacyclic triterpenoid first isolated from licorice (*Glycyrrhiza uralensis*) in the 1930s [142,143]. Glycyrrhetinic acid itself exhibits a broad range of pharmacological properties, including anti-inflammatory, hepatoprotective, antiviral, and anticancer activities, and has served as a versatile scaffold for chemical derivatization [144,145]. Building on this framework, Kang and co-workers synthesized a novel thiirane-modified derivative, 2β,3β-epithio-18β-glycyrrhetinic acid (**71**). This compound displayed pronounced anticancer activity, demonstrating that introduction of an epithio group can significantly enhance the cytotoxic potential of triterpenoid and steroidal molecules [146].

To summarize, these findings highlight epithio steroids as a chemically and biologically intriguing class of synthetic steroids. Their strong antiproliferative effects, coupled with tunable structural features, suggest that further exploration of thiirane-modified steroid frameworks may yield valuable leads for the development of next-generation anticancer agents.

## 10. Halogenated Steroids and Their Biological Activities

Halogenated natural products represent one of the most rapidly expanding and chemically intriguing classes of bioactive metabolites in contemporary organic and medicinal chemistry. Over the past three decades, systematic investigations—most notably those led by Professor Gordon Gribble (Dartmouth College)—have revealed an extraordinary diversity of halogen-containing compounds in nature. To date, more than 6000 halogenated metabolites have been identified from microorganisms, fungi, plants, marine algae, marine invertebrates, and even higher animals, underscoring the widespread biological utilization of halogen chemistry [147].

The biosynthetic incorporation of halogen atoms—fluorine, chlorine, bromine, and iodine—into organic frameworks is most commonly mediated by haloperoxidases and related halogenating enzymes. These enzymes are prevalent in marine organisms, fungi, algae, and bacteria, where they catalyze site-selective halogenation using halide ions and reactive halogen species. From a pharmacological perspective, halogen substitution often exerts profound effects on molecular properties, including increased lipophilicity, altered electronic distribution, enhanced metabolic stability, and improved receptor binding affinity. As a result, halogenated steroids frequently display enhanced or qualitatively altered biological activity compared with their non-halogenated counterparts.

In parallel with natural product discovery, the deliberate synthesis of halogenated steroids has become an important strategy in medicinal chemistry, endocrinology, and sports pharmacology. Many synthetic halogenated steroids have been designed to improve anabolic potency, prolong biological half-life, or modulate selectivity toward steroid hormone receptors. These compounds are of both clinical and biochemical interest, although some have also been misused in athletic performance enhancement [148,149].

### 10.1. Fluorinated Steroids and Their Biological Activity

Naturally occurring fluorinated organic compounds are exceptionally rare, reflecting the limited bioavailability of fluorine and the high energetic barrier associated with C–F bond formation. The first naturally occurring fluorinated metabolite, fluoroacetic acid, was identified in 1943 by Marais from the South African plant *Dichapetalum cymosum*. Subsequent work by O’Hagan and co-workers demonstrated that several *Dichapetalum* species—particularly *D. braunii*—accumulate extraordinarily high levels of fluoroacetate, reaching concentrations of up to 8000 mg/kg dry weight in young leaves and seeds. Additional natural sources include *Spondianthus preussi* and the Australian shrub *Acacia georginae*, where fluoroacetate levels of 250–400 mg/kg have been reported [150,151,152,153].

**Figure 17:**
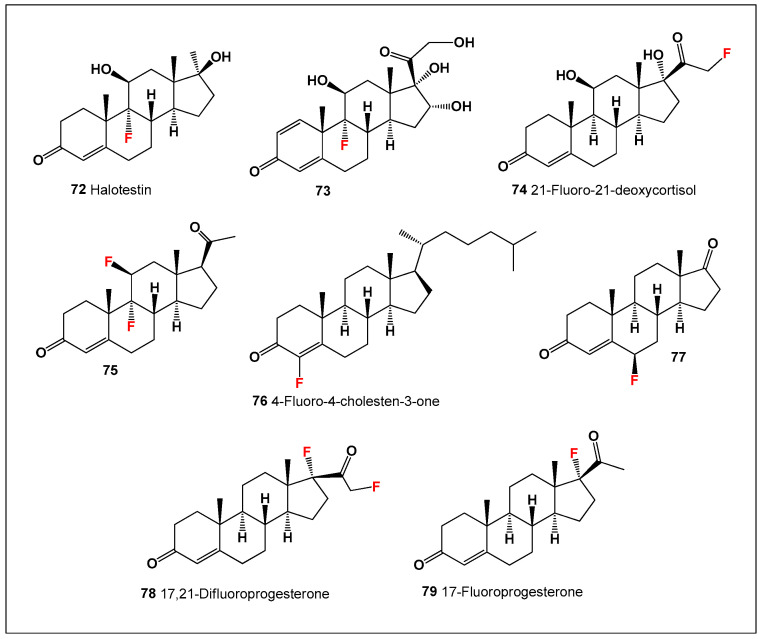
Representative bioactive fluorinated synthetic steroids (**72**–**79**) illustrating the structural diversity of this class and their broad pharmacological profiles. The depicted compounds exhibit pronounced anti-inflammatory, antiallergic, antiasthmatic, antiarthritic, and antineoplastic activities, reflecting the impact of fluorine substitution on steroidal potency, metabolic stability, and receptor interactions. Comparative activity of steroids is shown in Figure 18.

Despite the existence of naturally fluorinated metabolites, fluorinated steroids have never been identified in nature. All known fluorosteroids are synthetic and were developed primarily to exploit the unique physicochemical effects of fluorine substitution. Introduction of fluorine into steroidal frameworks often enhances metabolic stability by inhibiting oxidative degradation, increases receptor affinity, and modulates hormonal activity. Consequently, fluorosteroids (Figure 17) have been extensively investigated for anti-inflammatory, androgenic/anabolic, and anticancer properties, and several have found clinical application or experimental use [154,155,156,157,158].

**Figure 18:**
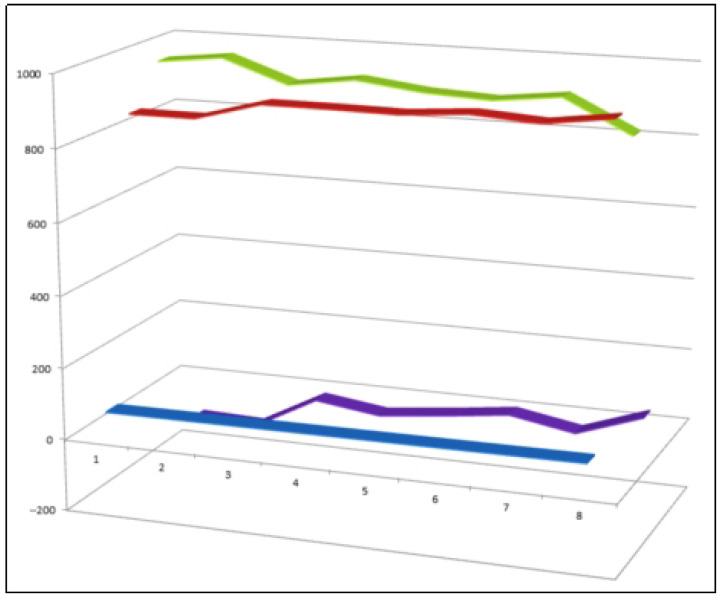
Three-dimensional activity profile of eight fluorinated steroids (**72**–**79**) illustrating the comparative balance between anti-inflammatory (green) and anticancer (red) effects. For most compounds, anti-inflammatory activity predominates and falls within the strong activity range (values of 900–980). Steroid **79** represents an exception, showing reduced anti-inflammatory potency and a shifted activity balance. This visualization highlights the role of fluorine incorporation in enhancing anti-inflammatory efficacy while maintaining relevant antineoplastic potential.

One of the most extensively studied fluorinated steroids is halotestin (**72**), a 17α-alkylated fluorinated testosterone derivative synthesized in the late 1950s [159,160]. Halotestin is a potent anabolic–androgenic steroid (AAS) and a strong inhibitor of 11β-hydroxysteroid dehydrogenase type 2, a key enzyme involved in glucocorticoid metabolism. Beyond its endocrine effects, halotestin has demonstrated significant anticancer activity, particularly against breast cancer cell lines, highlighting the potential of fluorinated steroids as antineoplastic agents [161,162,163].

In addition to halotestin, a wide range of structurally diverse fluorinated steroids (**73**–**79**) has been synthesized across multiple laboratories worldwide. These compounds collectively illustrate how strategic fluorine incorporation can fine-tune steroidal activity profiles, balancing anabolic, anti-inflammatory, and anticancer effects while improving pharmacokinetic behavior [155,156,164,165,166,167,168,169].

### 10.2. Chlorinated Steroids and Their Biological Activity

Chlorinated steroids occur in both natural and synthetic forms and collectively exhibit a wide spectrum of biological activities. Naturally occurring chlorinated metabolites have been isolated from terrestrial organisms and numerous marine species, whereas synthetic chlorinated steroids have been extensively developed for pharmacological research, clinical applications, and performance enhancement [170,171,172]. Chlorine substitution within the steroidal framework significantly alters physicochemical properties—such as lipophilicity, metabolic stability, and receptor binding—which in turn modulates biological potency and selectivity.

**Figure 19:**
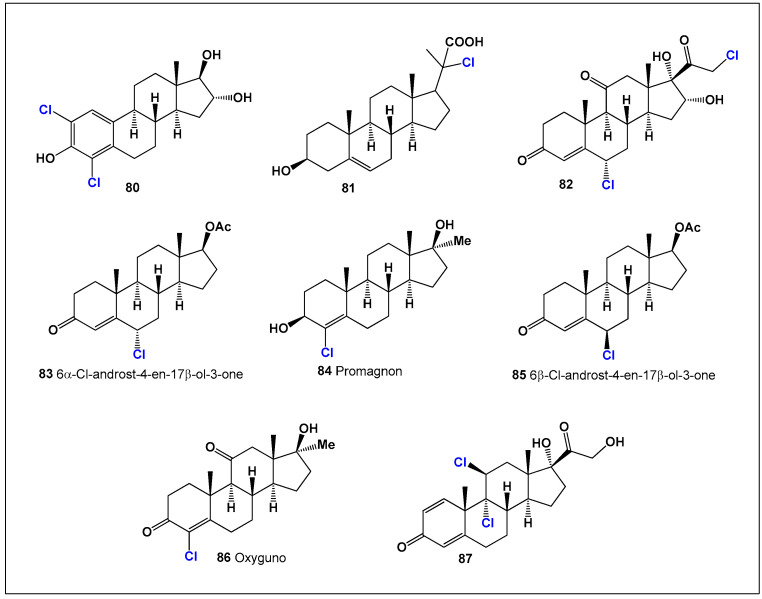
Representative bioactive chlorine-containing synthetic steroids discussed in Section 10.2. These compounds (**80**–**87**) illustrate the structural diversity of chlorinated androgenic and estrogenic steroids developed for pharmacological and biomedical research. Chlorine substitution at different positions within the steroidal framework significantly influences receptor affinity, metabolic stability, and the balance between anabolic, anti-inflammatory, and antineoplastic activities. Quantitative comparisons of their biological activities are summarized in Figure 20.

Early work by Nakamura and co-workers led to the synthesis of several chloro-estrogens, including 2,4-dichloroestriol (**80**; Figure 19), which exhibited measurable estrogenic activity [173]. Additional chlorinated steroids—**81**, **82**, and **87**—were prepared by multiple research groups during the mid-20th century and contributed to the foundational development of halogenated steroid chemistry, particularly in the context of hormone analog design [174,175,176,177].

**Figure 20:**
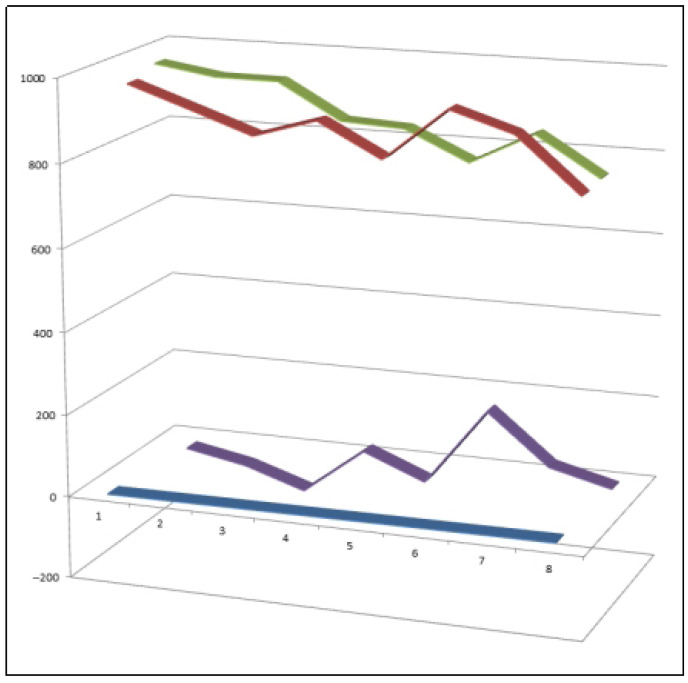
Three-dimensional activity profile of eight chlorinated synthetic steroids (**80**–**87**) illustrating the relationship between anti-inflammatory (green) and anticancer (red) activities. For most compounds, anti-inflammatory effects predominate, with activity values ranging from 900 to 980, whereas anticancer activity generally falls within the 770–950 range. Steroids **83**, **85**, and **86** deviate from this trend, displaying relatively enhanced anticancer activity. This visualization highlights how chlorine substitution modulates the balance between inflammation suppression and tumor-related bioactivity, emphasizing key structure–activity relationships within this steroid class.

Chlorinated androgens have attracted particular attention due to their pronounced anabolic activity. 6-Chloro-androst-4-en-3-one-17β-ol (hexadrone, **83**) is regarded as a new-generation androgenic compound designed to promote substantial increases in muscle mass and strength. As a potent prohormone, hexadrone reportedly exhibits a high anabolic-to-androgenic ratio of approximately 300:1, with minimal water retention, characteristics that have made it especially appealing in sports performance contexts [178].

Similarly, 3-chloro-17-methylandrostenediol (promagnon, **84**) is a methylated testosterone analogue developed to enhance muscle hypertrophy, vascularity, and muscular density. Chlorine substitution combined with 17α-methylation improves oral bioavailability and anabolic efficacy, illustrating how strategic halogenation can optimize pharmacokinetic properties [179].

The stereochemical influence of chlorine substitution is exemplified by 6β-chloro-androst-4-en-17β-ol-3-one (**85**), an epimer of hexadrone that displays similar but attenuated anabolic effects, underscoring the importance of C-6 configuration in modulating biological activity [116].

Another historically significant compound is 4-chloro-17α-methyl-etioallochol-4-ene-17β-ol-3,11-dione (oxyguno, **86**), a potent anabolic steroid developed in the German Democratic Republic during the 1960s. Despite possessing only ~7% of the androgenic activity of testosterone, oxyguno exhibits anabolic potency exceeding 850%, and it became one of the agents employed in systematic state-sponsored athletic doping programs of that era [180,181].

These examples illustrate the profound pharmacological impact of chlorine incorporation into steroidal scaffolds. Chlorine substitution not only enhances metabolic stability and receptor affinity but also enables fine-tuning of anabolic versus androgenic effects. These structure–activity relationships highlight the broader relevance of halogenation strategies in steroid-based drug design, with implications extending beyond endocrinology to inflammation, oncology, and metabolic disease research.

### 10.3. Chlorinated Plant Steroids and Their Biological Activities

Withanolides constitute a large and structurally diverse class of polyoxygenated C_28_ steroidal lactones that have been used for more than three millennia in Ayurvedic, Unani, and other traditional medical systems throughout Asia. In traditional Chinese medicine, plant extracts enriched in withanolides are widely recognized for their adaptogenic, diuretic, anti-inflammatory, anxiolytic, cytotoxic, antitussive, and immunomodulatory effects. Contemporary pharmacological studies have substantiated these traditional claims and revealed that withanolides modulate multiple inflammatory and stress-related signaling pathways, including NF-κB, JAK/STAT, AP-1, PPARγ, Hsp90, Nrf2, and HIF-1, thereby exerting pleiotropic biological effects [182,183,184,185].

To date, more than one thousand withanolide-type metabolites have been identified, primarily from species belonging to the genera *Withania*, *Physalis*, and *Jaborosa*. Although halogenation is generally rare in plant secondary metabolism, several chlorinated withanolides and related plant steroids have been isolated and shown to possess notable biological activities, including cytotoxic, anti-inflammatory, antioxidant, and cytostatic effects [184,185]. These rare metabolites significantly expand the chemical space of plant-derived steroids and provide valuable insight into the impact of halogen substitution on biological function.

**Figure 21:**
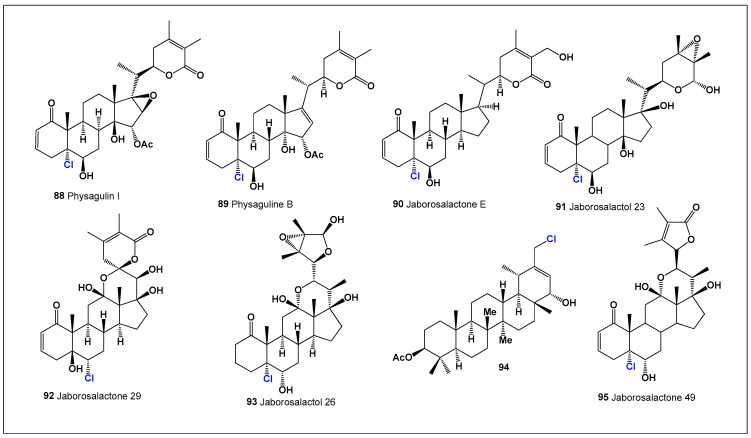
Representative bioactive chlorine-containing steroids and triterpenoids (**88**–**95**) isolated from terrestrial plant sources, including chlorinated withanolides and taraxastane-type derivatives. These compounds illustrate the structural diversity introduced by chlorine substitution within plant steroidal frameworks. Comparative biological activity profiles for these metabolites are summarized in Figure 22.

Among chlorinated withanolides, physagulin I (**88**, Figure 21), a 14β-hydroxy-withanolide bearing an α-oxygenated substituent at C-15, was isolated from *Physalis* species [186], while physaguline B (**89**) was obtained from *Physalis angulata* [187]. Jaborosalactone E (**90**) was first identified in the leaves of *Jaborosa integrifolia* (Solanaceae) [188] and was later detected in *Acnistus breviflorus*, where it exhibited cytostatic activity in cell-based assays [184]. Another structurally related metabolite, the 14β-hydroxywithanolide jaborosalactol 23 (**91**), was isolated from *Jaborosa bergii* [189].

**Figure 22:**
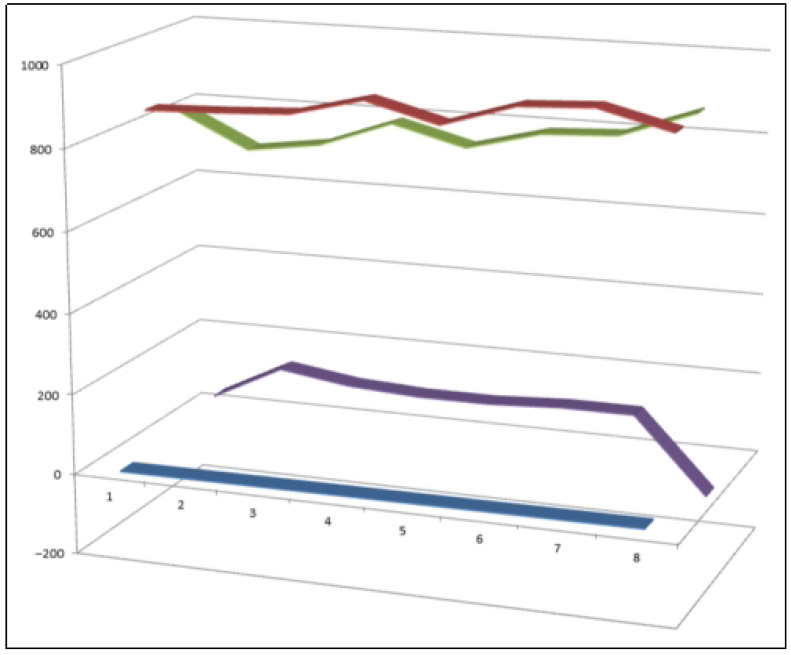
Three-dimensional activity plot illustrating the relationship between anticancer (red) and anti-inflammatory (green) activities for chlorine-containing plant-derived steroids and triterpenoids (**88**–**95**). The plot reveals a general predominance of antineoplastic activity over anti-inflammatory effects, with anticancer activity values ranging from 860 to 920, while anti-inflammatory activity values fall within the moderate-to-strong range (720–820). This distribution highlights the tendency of chlorine substitution in plant steroids to preferentially enhance cytotoxic and antiproliferative properties.

Further structural diversity is illustrated by a unique withanolide (**92**) isolated from *Jaborosa rotacea*, characterized by a hemiketal bridge formed from former ketone groups at C-12 and C-22. This transformation generates a six-membered ring bearing a β-oriented hydroxyl group at C-12 and a spiroketal configuration at C-22, representing an uncommon rearrangement within the withanolide family [190]. In *Jaborosa parviflora*, a chlorinated 24,25-epoxy-γ-lactol (**93**) was detected [191], while the chlorohydrin jaborosalactone 49 (**95**) was isolated from *J. caulescens* var. *bipinnatifida* [192] and *J. laciniata* [193].

Beyond withanolides, chlorinated plant steroids have also been identified in other botanical taxa. Extraction of the aerial parts of *Tolpis proustii* and *T. lagopoda* (La Gomera, Canary Islands) yielded 30-chloro-3β-acetoxy-22α-hydroxy-20(21)-taraxastene (**94**), a chlorinated taraxastane-type triterpenoid. While crude extracts showed antioxidant activity in DPPH and ABTS assays, compound **94** exhibited cytotoxicity against human myeloid leukemia cell lines K-562 and K-562/ADR, underscoring the biological relevance of chlorine substitution in plant triterpenes [194]. These examples demonstrate that chlorinated plant steroids, although rare, significantly enrich the structural and functional diversity of halogenated natural products and often display enhanced anticancer, anti-inflammatory, or antioxidant properties relative to their non-halogenated counterparts.

Particularly intriguing data have been reported for withanolide D chlorohydrin (**96**), a rare chlorinated withanolide isolated from *Withania somnifera* (ashwagandha) [195]. *W. somnifera* is a well-established adaptogenic plant extensively used in Ayurvedic medicine for centuries to enhance stress tolerance and overall physiological resilience [196]. For compound **96**, both computational (PASS-based) and experimental analyses identified five major biological activity categories contributing to its pharmacological profile. The most prominent predicted activities were neuroprotective and neurodegenerative disease-modulating effects, including treatment of neurodegenerative disorders (22.31%), Alzheimer’s disease-related activity (21.81%), and antiparkinsonian effects (19.10%), followed by antineoplastic (18.52%) and anti-inflammatory activities (18.24%) [195,196].

The predominance of neurological activities distinguishes withanolide D chlorohydrin from most other known withanolides, which typically display primarily antitumor or anti-inflammatory profiles. In this exceptional case, compound **96** exhibits a rare multi-target neuropharmacological spectrum, suggesting potential relevance for neurodegenerative disorders such as Alzheimer’s and Parkinson’s diseases. Although its antineoplastic and anti-inflammatory effects are comparatively less dominant, they remain biologically meaningful and may act synergistically with neuroprotective mechanisms.

**Figure 23:**
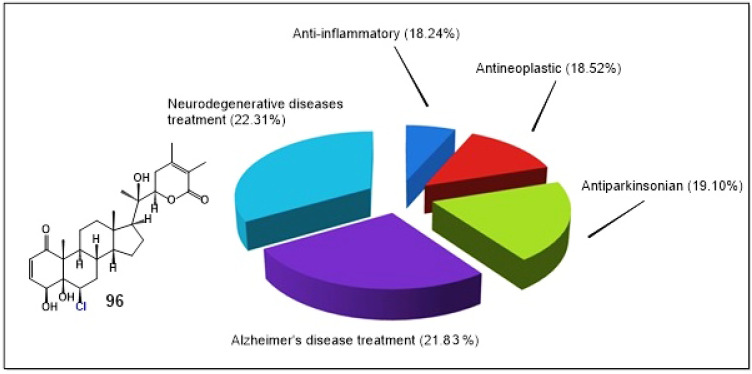
Percentage distribution of predicted and experimentally supported biological activities of withanolide D chlorohydrin (**96**) isolated from *Withania somnifera*. The activity profile is normalized to 100% and illustrates the relative contributions of neuroprotective, antineurodegenerative (Alzheimer’s and Parkinson’s disease-related), anticancer, and anti-inflammatory effects. Notably, neurological activities represent the dominant component of the predicted bioactivity spectrum, distinguishing this compound from most other withanolides, which typically exhibit primarily antitumor or anti-inflammatory properties. Although clinical validation is required, this unique multi-target activity profile highlights withanolide D chlorohydrin as a promising lead structure for further pharmacological and preclinical investigation in neurodegenerative disorders.

Figure 23 presents the normalized percentage distribution of the predicted and experimentally supported biological activities of withanolide D chlorohydrin (**96**), illustrating the balanced yet neurologically biased activity profile of this structurally unique chlorinated withanolide.

### 10.4. Chlorinated Marine Steroids and Their Biological Activities

Marine organisms—including macroalgae, sponges, corals, and echinoderms—constitute one of the richest reservoirs of structurally novel secondary metabolites, many of which exhibit pronounced anti-inflammatory, cytotoxic, and anticancer activities [197,198,199,200,201,202]. Within this vast chemical space, chlorinated marine steroids represent an exceptionally rare yet pharmacologically powerful subgroup. These compounds are often characterized by highly unusual carbon skeletons, extensive halogenation, and strong bioactivity, reflecting both unique marine biosynthetic pathways and the selective pressures of marine chemical ecology.

**Figure 24:**
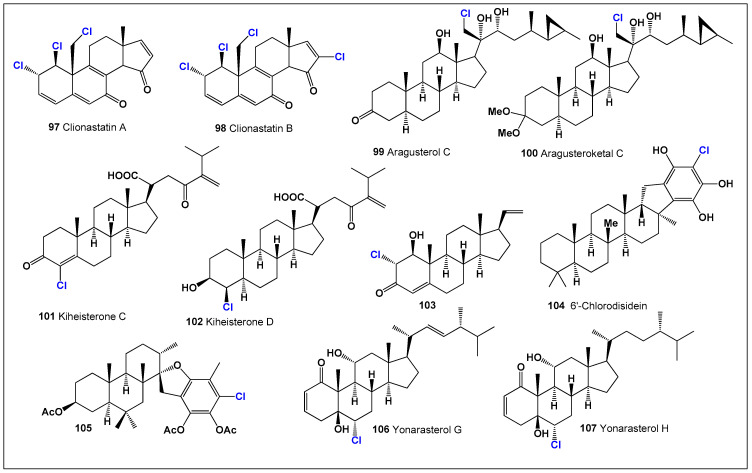
Representative chlorinated steroids and triterpenoids (**97**–**107**) isolated from marine algae and marine invertebrates, including sponges, corals, and echinoderms. These metabolites display highly unusual carbon skeletons and diverse chlorine substitution patterns that are characteristic of marine halogenated biosynthesis and are frequently associated with pronounced cytotoxic, antiproliferative, and anti-inflammatory activities. Comparative activity of steroids is shown in Figure 25.

Among the most remarkable examples are the highly cytotoxic chlorinated androstane derivatives clionastatin A (**97**, Figure 24) and clionastatin B (**98**), isolated from the burrowing sponge *Cliona nigricans*. These molecules possess unprecedented tri- and tetrachlorinated androstane frameworks and represent the first naturally occurring polyhalogenated steroids identified in any biological system—marine or terrestrial—as well as the first halogenated androstanes discovered in nature [203]. Their discovery fundamentally expanded the known limits of natural steroid halogenation and underscored the capacity of marine organisms to generate chemically extreme metabolites with potent biological effects.

**Figure 25:**
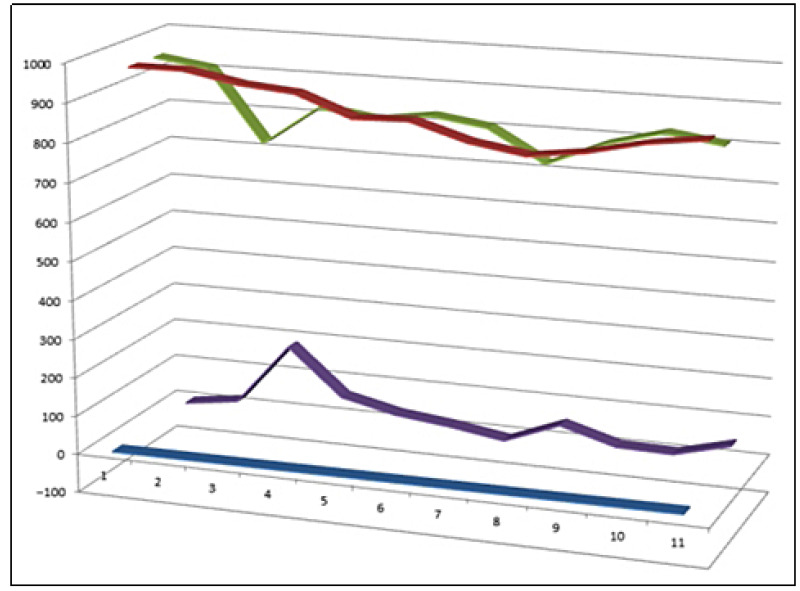
Three-dimensional activity profile of chlorinated natural steroids (**97**–**107**) isolated from marine organisms. The plot illustrates the relative balance between anti-inflammatory activity (green) and anticancer activity (red) across this compound set. Most metabolites exhibit comparable potency in both biological domains, supporting the concept of dual-action steroidal frameworks. A notable reduction in anti-inflammatory activity is observed for steroid **99**, consistent with its strong bias toward cytotoxic and antitumor effects.

Another notable chlorinated marine steroid, aragusterol C (**99**), was isolated from an Okinawan sponge of the genus *Xestospongia*. Aragusterol C demonstrated strong in vitro antiproliferative activity against human KB carcinoma cells and exhibited significant in vivo antitumor efficacy against L1210 leukemia in murine models [204]. A structurally related metabolite, aragusteroketal C (**100**), containing a dimethylketal functionality, was isolated from the same sponge and showed extraordinary cytotoxic potency, with an IC_50_ value of only 4 ng/mL against KB tumor cells [205]. This exceptional activity places aragusteroketal C among the most potent marine-derived steroidal cytotoxins reported to date.

Further expanding this class, cytotoxic chloro-ketosteroids designated kiheisterones C (**101**) and D (**102**) were isolated from the marine sponge *Strongylacedon* sp. collected in Hawaiian waters. These metabolites highlight the continued discovery of structurally diverse chlorinated steroids from sponges and reinforce the role of chlorine substitution in enhancing cytotoxic and antiproliferative properties [206].

Chlorinated marine steroids are not limited to sponges. The Mediterranean red starfish *Echinaster sepositus* produces the chlorinated sterol (3β,5α,22R,23S)-22-chlorocholesta-8,14-diene-3,23-diol (**103**), illustrating that echinoderms also contribute to the biosynthesis of halogenated sterols with potential biological relevance [207]. In addition, a structurally unique pentacyclic saturated sesterpene conjugated to a hydroxyhydroquinone moiety, 6′-chlorodisidein (**104**), was isolated as a sodium–calcium disulfate salt from the sponge *Disidea pallescens*, further emphasizing the chemical novelty achievable through marine halogenation pathways [208].

Marine algae and soft corals also yield chlorinated steroidal metabolites. The brown alga *Stypopodium flabelliforme* afforded chlorinated stypotriol triacetate (105) from its dichloromethane extract [209], while the eastern Pacific octocoral *Carijoa multiflora* produced two rare chloro-pregnane steroids (**106** and **107**), whose biological activity profiles are summarized in Figure 22 [210].

These chlorinated marine steroids exemplify the extraordinary structural diversity, biosynthetic ingenuity, and potent biological activities characteristic of marine halogenated metabolites. Their strong cytotoxic and antitumor effects—often exceeding those of non-halogenated analogues—underscore the critical role of chlorine substitution in modulating steroidal bioactivity. As such, chlorinated marine steroids represent highly promising lead structures for anticancer drug discovery and provide valuable insights into structure–activity relationships linking halogenation, molecular architecture, and biological function.

### 10.5. Brominated Steroids and Their Biological Activity

Brominated natural products are biosynthesized predominantly by marine organisms, particularly sponges and marine macroalgae, where bromide ions are abundant and haloperoxidase-mediated halogenation is widespread [211,212,213,214,215]. In addition to marine environments, brominated metabolites have also been detected in extremophilic lichens of the genus *Acarospora*, inhabiting hypersaline salt mountains near the Dead Sea, highlighting the adaptive role of bromination under chemically extreme conditions [216,217,218,219,220,221]. In contrast, brominated secondary metabolites are essentially absent from terrestrial plants and higher animals, underscoring their status as distinctive chemical signatures of marine and extremophilic biosystems.

Within this broader class of halometabolites, brominated steroids represent an exceptionally rare subgroup of marine lipids. To date, all naturally occurring brominated steroids have been isolated exclusively from marine sponges, reflecting both the specialized biosynthetic capacity of these organisms and the ecological pressures of the marine environment. The most prominent examples are the structurally related C-nor-D-homosteroids nakyterpiosinone and nakyterpiosin, isolated from the sponge *Terpios hoshinota*. These metabolites possess highly unusual rearranged steroidal skeletons combined with bromine substitution, a structural motif rarely encountered in steroid chemistry.

Biologically, nakyterpiosinone and nakyterpiosin exhibit potent anticancer activity, particularly against tumor cell lines that are resistant to conventional antimitotic agents. Mechanistic studies indicate that these compounds interfere with aberrant activation of the Hedgehog signaling pathway, a key developmental pathway frequently hijacked in cancer to promote uncontrolled proliferation, survival, and drug resistance [222,223]. Their ability to target this pathway positions brominated marine steroids as promising lead structures for the development of anticancer agents aimed at refractory and signaling-driven malignancies.

**Figure 26:**
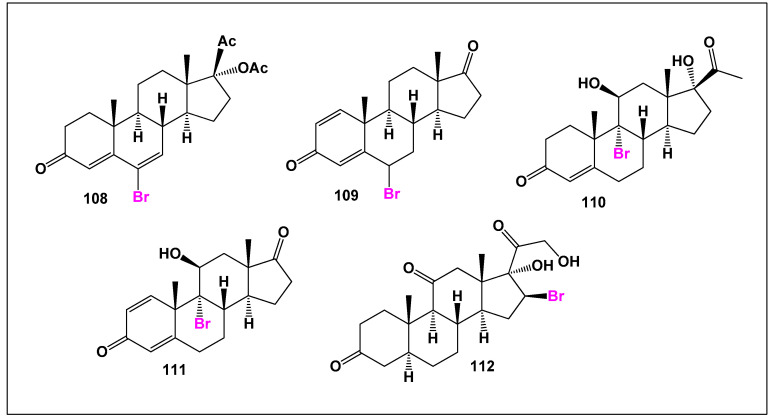
Representative brominated synthetic steroids, illustrating structural diversity and bromine substitution patterns. Their comparative biological activities are summarized in Figure 27.

Despite their pronounced antiproliferative and cytotoxic effects, natural brominated steroids (Figure 26) have not yet been systematically evaluated for anti-inflammatory activity. This gap in knowledge represents an important opportunity for future pharmacological research, particularly given the emerging paradigm that inflammation and cancer are mechanistically interconnected. Exploration of anti-inflammatory endpoints may reveal additional therapeutic dimensions or dual-action potential within this rare class of marine steroids.

**Figure 27:**
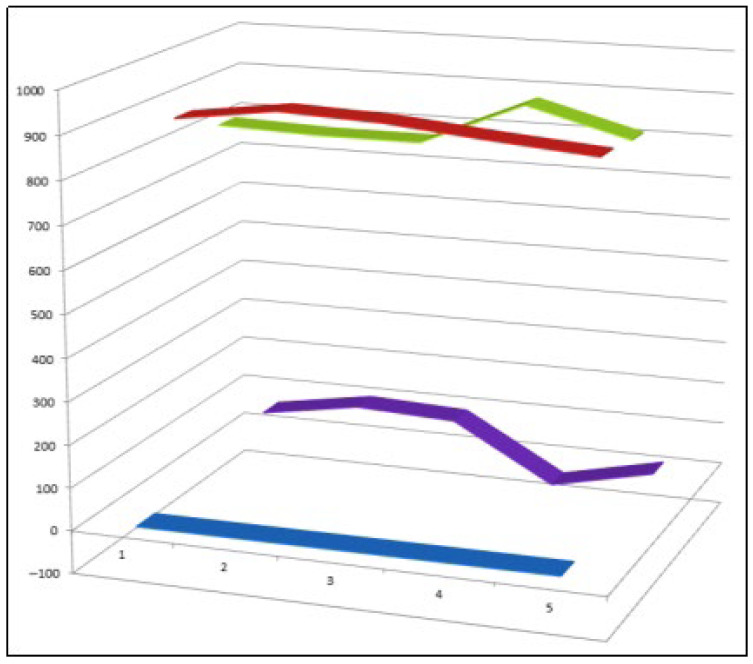
Three-dimensional activity profile of brominated synthetic steroids (**108**–**112**)**,** showing the relationship between anti-inflammatory activity (green) and anticancer activity (red). Overall, these compounds exhibit a relatively balanced dual-activity profile, with steroids **111** and **112** displaying enhanced anti-inflammatory activity, suggesting that bromine substitution may modulate inflammatory signaling in a structure-dependent manner.

### 10.6. Iodinated Steroids and Their Biological Activity

Natural iodinated secondary metabolites are produced almost exclusively by marine macroalgae and marine invertebrates, where the exceptionally high bioavailability of iodide in seawater enables its incorporation into a wide range of organic scaffolds, including lipids, fatty acids, peptides, terpenoids, and phenolic compounds [211,217,224,225,226,227]. In marine systems, iodine is believed to play important roles in oxidative stress regulation, chemical defense, and halogen cycling. Despite this abundance of iodinated metabolites in marine organisms, iodinated steroids have not been identified as natural products, suggesting either biosynthetic constraints or rapid metabolic instability of iodine-substituted steroidal frameworks in vivo.

Nevertheless, synthetic iodinated steroids represent a modest but chemically intriguing class, with more than 100 compounds reported to date. Compared with fluorinated, chlorinated, and brominated steroids, the pharmacology of iodinated analogues remains relatively underexplored, largely due to their lower metabolic stability and weaker receptor affinity—features attributable to iodine’s large atomic radius, lower electronegativity, and high polarizability.

Early and influential studies by Fried [228] provided clear structure–activity relationships for 9α-halogenated-11-hydroxyprogesterones, demonstrating a pronounced dependence of biological potency on the nature of the halogen substituent. In rat models measuring inhibition of hepatic glycogen biosynthesis, glucocorticoid activity increased in the order:

I < Br < Cl < F,

with relative activity values of 0.1 (iodo), 0.3 (bromo), 4.7 (chloro), and 10.7 (fluoro), respectively. These results elegantly illustrate how halogen electronegativity, steric demand, and bond strength collectively govern steroid–receptor interactions, metabolic resistance, and downstream biological responses. In particular, the comparatively weak activity of iodinated steroids reflects steric hindrance and suboptimal hydrogen-bonding interactions within the glucocorticoid receptor binding pocket.

**Figure 28:**
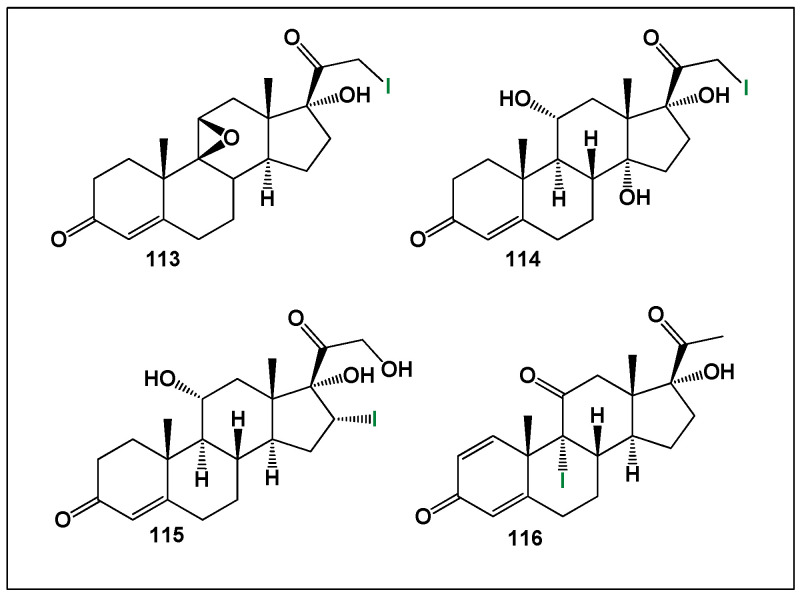
Representative bioactive synthetic iodinated steroids (**113**–**116**) selected from the limited but pharmacologically informative class of iodine-substituted steroid analogues. These compounds illustrate the structural diversity achievable through iodine incorporation at different positions of the steroidal framework. Despite the general scarcity and comparatively lower potency of iodinated steroids relative to fluorinated and chlorinated analogues, compounds **113**–**116** display measurable anti-inflammatory and antitumor activity, highlighting the potential of iodine substitution to modulate biological responses. Quantitative activity profiles for these steroids are presented in Figure 29.

From a synthetic perspective, several iodinated pregnane derivatives have been prepared to probe these structure–activity trends. Two 21-iodo-20-ketopregnanes (**113** and **114**, Figure 28) were synthesized using improved iodination methodologies developed by Heinrich [229] and Stork and co-workers [230]. These compounds served as early model systems for evaluating the effects of iodine substitution at the terminal side chain on steroidal bioactivity.

**Figure 29:**
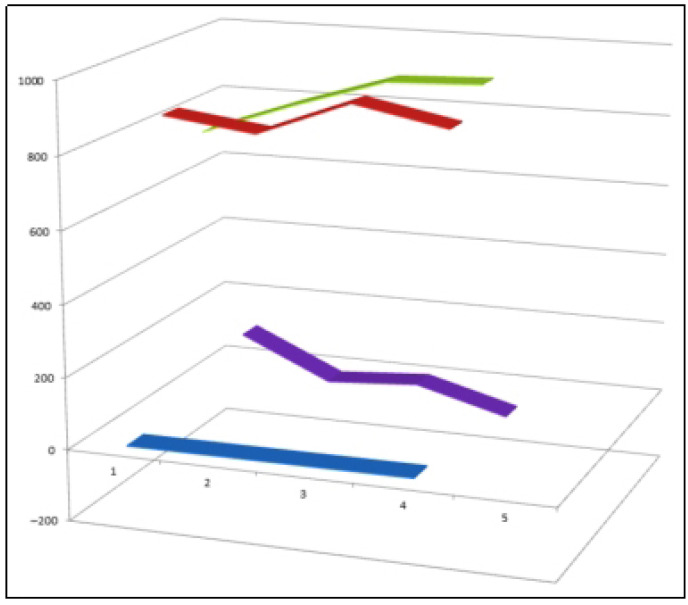
Three-dimensional activity profile of iodinated steroids (**113**–**116**) comparing anti-inflammatory (green) and antitumor (red) activities. Blue bars represent individual steroid numbering, while purple bars indicate the calculated difference between anticancer and anti-inflammatory effects for each compound. Activity values in the range of 800–900 correspond to strong biological activity, 700–800 indicate moderate activity, and values below 700 represent low activity. The visualization demonstrates that, although iodinated steroids generally exhibit lower potency than other halogenated analogues, select compounds retain balanced or moderately strong dual activity, underscoring the nuanced role of iodine substitution in shaping steroid–receptor interactions and downstream pharmacological effects.

In addition, a 16-iodosteroid (**115**) was reported to exhibit measurable anti-inflammatory and glucocorticoid activity, indicating that strategic placement of iodine within the steroid nucleus can partially compensate for its intrinsic steric disadvantages [231]. Fried [228] further described the synthesis of another iodine-containing steroid (**116**), contributing to the foundational chemical and pharmacological understanding of this understudied class.

Although iodinated steroids generally display lower potency than their fluorinated or chlorinated counterparts, their high polarizability and distinctive electronic properties may offer advantages in niche applications, such as radiolabeling, mechanistic receptor studies, or the design of prodrugs with controlled metabolic activation. Continued exploration of iodinated steroid analogues—particularly in the context of dual anti-inflammatory and anticancer activity—may uncover previously unrecognized pharmacological niches or complementary therapeutic roles.

## 11. Steroid Phosphate Esters and Their Biological Activity

Steroid phosphate esters comprise a specialized class of steroidal derivatives in which one or more phosphate groups are covalently linked to the steroid backbone via ester bonds. This modification profoundly alters the physicochemical and biological properties of the parent steroid. In particular, phosphorylation increases molecular polarity, aqueous solubility, and metabolic stability, while also influencing membrane permeability, intracellular trafficking, and tissue selectivity. These features have positioned steroid phosphate esters as attractive scaffolds in drug design, especially for applications requiring improved pharmacokinetic profiles or targeted biological activity [59,232].

From a biological perspective, phosphate esters play indispensable roles in cellular signaling, energy metabolism, and hormonal regulation. In steroid biosynthesis, phosphorylated intermediates participate in key enzymatic steps, including the conversion of cholesterol to pregnenolone, underscoring the physiological relevance of transient steroid phosphorylation. In medicinal chemistry, the deliberate introduction of phosphate groups has been widely exploited to enhance oral bioavailability, prolong circulation time, reduce nonspecific toxicity, and improve intracellular delivery of steroid-based therapeutics. As a result, phosphorylated steroids have found applications across diverse therapeutic areas, including endocrinology, cardiology, oncology, and inflammatory disease management [233,234,235,236].

### 11.1. Discovery of Natural Steroid Phosphate Esters

Until the early 1990s, steroid phosphate esters were widely regarded as exclusively synthetic constructs, with no confirmed occurrence in nature. This view was overturned by the discovery of the first naturally occurring steroid phosphate esters by Italian researchers at the University of Naples Federico II. These compounds were isolated from the deep-sea starfish *Tremaster novaecaledoniae*, collected at a depth of approximately 530 m off the coast of New Caledonia [237]. The metabolites, designated tremasterols A–C, are steroidoglycosides characterized by an unprecedented combination of functional groups, including a 3β-O-sulfate, a 6α-O-phosphate, and a 16β-O-acetyl substituent. This finding provided the first unequivocal evidence that phosphorylation can occur as a natural post-biosynthetic modification of steroids.

Subsequently, a second class of naturally occurring phosphated steroids—haplosamates A and B—was isolated from the marine sponge *Cribrochalina* sp., together with a minor secosteroid [238]. Haplosamate A possesses a uniquely decorated C_28_ sterol skeleton bearing both a sulfate group at C-3 and a methyl phosphate group at C-15, whereas haplosamate B contains two phosphate groups at C-7 and C-15. These complex substitution patterns suggest the involvement of specialized enzymatic machinery capable of regioselective phosphorylation and sulfation in marine organisms. Despite their striking structural novelty, none of these naturally occurring phosphated steroids demonstrated pronounced anti-inflammatory activity, indicating that phosphorylation alone is not sufficient to confer anti-inflammatory potency.

An additional and unexpected observation was the detection of steroid phosphate esters in the eggs of the desert locust *Schistocerca gregaria* [239]. Although the precise biosynthetic origin and physiological role of these metabolites remain unclear, their presence suggests that steroid phosphorylation may occur in specific developmental stages or under particular environmental or metabolic conditions. Collectively, these findings challenge earlier assumptions and indicate that steroid phosphate esters, while rare, do occur naturally across diverse biological systems.

### 11.2. Ouabain Phosphate Esters

**Figure 30:**
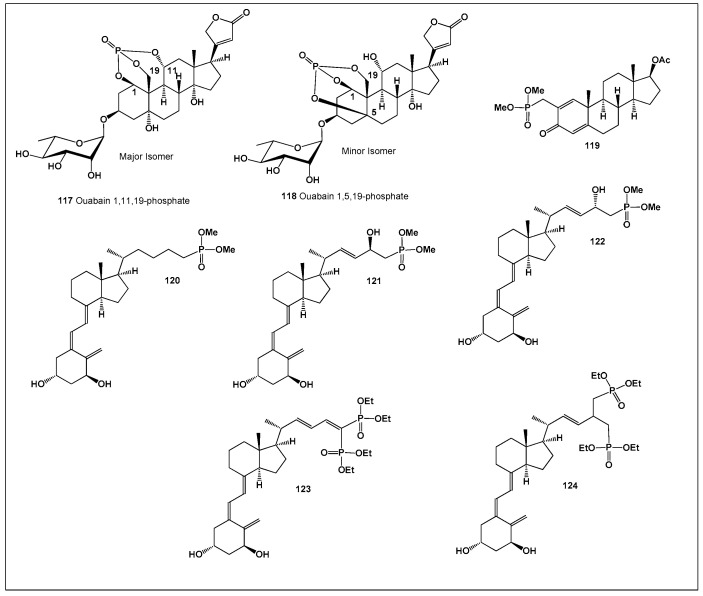
Representative bioactive natural sterol phosphate esters and synthetic phosphonosteroids (**117**–**124**) illustrating the structural diversity of phosphorus-containing steroidal derivatives. The figure includes naturally occurring sterol phosphates isolated from marine invertebrates (e.g., tremasterols and haplosamates), phosphate esters of cardiac glycosides (ouabain derivatives), and fully synthetic steroid phosphonates and bisphosphonates developed for therapeutic applications. The incorporation of phosphate or phosphonate groups markedly increases molecular polarity and influences membrane transport, metabolic stability, and target selectivity, thereby modulating both anti-inflammatory and antitumor biological profiles. Comparative activity of steroids is shown in Figure 31.

In the late 1990s, the first phosphate ester derivatives of the cardiac glycoside ouabain—compounds **117** and **118** (Figure 30)—were isolated and structurally characterized [240,241]. Ouabain (also known as G-strophanthin) is a well-known steroidal cardiotonic agent originally derived from African plants such as *Acokanthera schimperi* and *Strophanthus gratus*. Historically employed as an arrow poison, ouabain later became clinically relevant due to its potent inhibition of the Na^+^/K^+^-ATPase, leading to elevated intracellular calcium levels and enhanced myocardial contractility [242,243,244,245].

**Figure 31:**
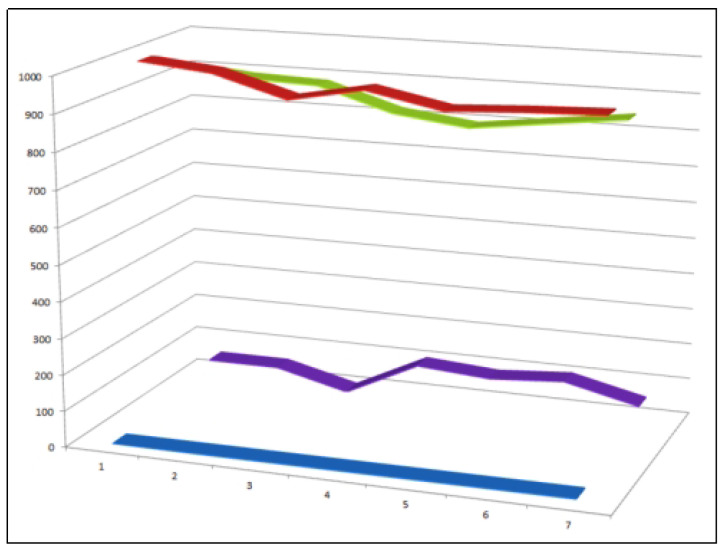
Three-dimensional activity profile of phosphorus-containing steroids (**117**–**124**) illustrating the quantitative relationship between antitumor and anti-inflammatory activities. Red bars represent calculated antineoplastic activity, green bars denote anti-inflammatory activity, blue bars indicate individual steroid numbering, and violet bars depict the numerical difference between anticancer and anti-inflammatory effects. The analysis demonstrates a clear predominance of antitumor activity across this class, with most compounds exhibiting activity values in the range of 850–980, corresponding to strong biological activity. These results suggest that phosphorylation and phosphonation of the steroidal framework preferentially enhance cytotoxic and antiproliferative effects, likely through improved cellular uptake and modulation of signaling pathways associated with apoptosis and tumor growth, while retaining moderate anti-inflammatory potential.

Beyond its cardiotonic effects, modern biochemical and pharmacological studies have revealed that ouabain and its analogues possess a surprisingly broad biological profile, including anticancer, antiviral, and anti-inflammatory activities. These effects are mediated not only through Na^+^/K^+^-ATPase inhibition but also via downstream modulation of intracellular signaling pathways, apoptosis, immune responses, and oxidative stress [246,247,248,249]. The discovery of ouabain phosphate esters further expanded the chemical space of this class, demonstrating that phosphorylation can be accommodated within complex cardiac glycosides without abolishing biological activity.

Importantly, ouabain phosphate esters represent some of the most biologically active members of the steroid phosphate ester family discussed in this review. Their dual anti-inflammatory and antineoplastic profiles, combined with improved physicochemical properties, underscore the potential of phosphate modification as a strategic tool for optimizing steroid-based therapeutics.

A broad range of synthetic phosphonosteroids (**119**–**124**) has been developed, many of which exhibit potent biological activity. Early work by Bravet and co-workers produced steroid phosphonates derived from estrone (e.g., compound **119**) [250,251].

Subsequently, several vitamin D phosphonate and bisphosphonate analogs (**120**–**124**) were synthesized [252]. These compounds act as potent inhibitors of osteoclast activation and are widely used in the treatment of osteoporosis, Paget’s disease, bone metastases associated with cancers such as breast and prostate carcinoma, and multiple myeloma.

The bisphosphonate-containing vitamin D analogs (**123** and **124**) are among the most clinically important steroid phosphonates due to their strong antiresorptive properties and antitumor activity [253,254,255]. Among the known steroid phosphate esters and phosphonosteroids, the strongest anti-inflammatory and antineoplastic activities were demonstrated by ouabain phosphate esters (**117**, **118**), and synthetic phosphorus-containing steroids (**119**–**124**).

These findings highlight the significant therapeutic potential of phosphate-modified steroids, particularly in oncology and inflammatory disease management.

## 12. Comparison of the Biological Activity of Natural and Synthetic Steroids

The biological activity of steroidal compounds is fundamentally determined by their chemical structure, a principle formalized within the structure–activity relationship (SAR) paradigm. Early qualitative observations by Brown and Fraser in the late nineteenth century demonstrated that even minor structural modifications could produce profound changes in biological response [256]. These insights were later systematized through the development of quantitative structure–activity relationships (QSARs) by Hansch and Fujita, establishing a predictive framework that remains central to medicinal chemistry, toxicology, and natural product research [257]. Together, SAR and QSAR concepts provide a rational basis for understanding how specific functional groups, stereochemistry, oxidation states, and scaffold rigidity modulate biological function [258].

Comparative analysis of natural and synthetic steroids reveals that the steroid nucleus represents a privileged and highly adaptable pharmacophore. A conserved polycyclic core supports extensive structural diversification, enabling interaction with a wide range of biological targets and signaling pathways. In natural steroids, evolutionary pressure has optimized functional group placement for ecological roles such as defense, signaling, and hormonal regulation. In contrast, synthetic modification—through halogenation, oxidation, epoxidation, nitrile or phosphate incorporation—has expanded this chemical space toward enhanced potency, selectivity, and metabolic stability. The present SAR/QSAR comparison demonstrates that both natural and synthetic steroids can converge on similar biological endpoints, including anti-inflammatory, antitumor, neuroprotective, and immunomodulatory activities, while differing in mechanistic emphasis and target engagement [259].

To systematically evaluate these relationships, biological activity prediction was performed using the PASS (*Prediction of Activity Spectra for Substances*) platform. PASS is a well-established in silico tool that predicts over 10,000 types of biological activities based solely on two-dimensional structural representations. These predictions encompass pharmacological effects, mechanisms of action, toxicological properties, enzyme and receptor interactions, and potential influences on gene expression. Because PASS does not require experimental input beyond structural information, it is particularly useful for assessing both isolated natural products and hypothetical or as yet unsynthesized steroid derivatives [260].

The predictive models implemented in PASS are derived from an extensive training set comprising more than one million biologically characterized compounds, including approved drugs, clinical candidates, and known toxic agents. Validation studies employing leave-one-out cross-validation have reported an average predictive accuracy of approximately 95%. Additional robustness tests using large independent datasets demonstrate that PASS maintains reasonable predictive performance even when a substantial portion of training data is removed, underscoring the stability of the underlying algorithm. Nonetheless, predictions are most reliable for compounds that fall within drug-like chemical space represented in the training set; results for structurally unprecedented molecules should be interpreted as exploratory hypotheses rather than definitive activity assignments [261,262].

Importantly, PASS predictions reflect the likelihood of biological relevance rather than confirmed functional outcomes. The platform may predict both agonistic and antagonistic interactions for the same target, indicating potential binding affinity rather than directionality of effect. Consequently, experimental validation remains essential to determine actual pharmacological behavior. Within this context, PASS serves not as a substitute for biological testing but as a prioritization and hypothesis-generation tool that guides rational selection of candidates for further study [262,263,264].

Among computational approaches applied to steroid research, PASS has proven particularly valuable for chemically complex and highly oxygenated steroids, whose redox sensitivity and functional group lability often complicate conventional SAR analysis. For both natural and synthetic steroids, PASS-based profiling enables rapid identification of dominant biological activity patterns, facilitates scaffold-level comparisons, and supports integrated experimental–computational strategies aimed at discovering multifunctional steroid-based therapeutics.

## 13. Conclusions

Steroids represent one of the most structurally versatile and biologically influential families of natural and synthetic metabolites. Across the diverse classes analyzed in this review—furanosteroids, neo-steroids, aromatic steroids, α,β-epoxy and peroxy steroids, cyanosteroids, nitro- and epithio steroids, halogenated derivatives, and steroid phosphate esters—a clear and recurring trend emerges: only a relatively small subset of known steroids exhibits simultaneous and potent anti-inflammatory and anticancer activity. However, when such dual activity is present, it is rarely coincidental and instead reflects shared molecular targets and convergent signaling pathways.

Chronic inflammation and tumorigenesis are tightly interconnected through overlapping regulatory networks that include oxidative stress responses, immune modulation, cytokine signaling, and the control of apoptosis and cell survival. Steroidal compounds capable of interacting with multiple nodes of these networks—particularly the NF-κB, PI3K/AKT, MAPK, JAK/STAT, and mitochondrial apoptotic pathways—are more likely to display complementary anti-inflammatory and antitumor effects. Comparative analysis across structural classes indicates that specific functional motifs, such as epoxide and peroxide bridges, nitrile and nitro substituents, thiirane rings, halogen atoms, and phosphate or phosphonate groups, play a decisive role in expanding molecular reactivity, target engagement, and biological breadth.

Marine organisms, fungi, and medicinal plants emerge as especially rich sources of chemically innovative steroidal frameworks, including polyhalogenated scaffolds, rearranged or seco-ring systems, spiroketals, and phosphorylated sterols. These rare architectures not only expand the known chemical space of steroids but also provide valuable insights into structure–activity relationships, demonstrating how subtle modifications of the steroid nucleus can shift biological balance toward inflammation suppression, cytotoxicity, or both. Synthetic modification of natural scaffolds further confirms that strategic incorporation of heteroatoms or charged groups can enhance cellular uptake, metabolic stability, and pathway selectivity.

In the aggregate, the compounds reviewed herein underscore the untapped therapeutic potential of steroidal metabolites as multifunctional agents. Furanosteroids, withanolides, halogenated marine steroids, epoxy- and peroxy-steroids, and phosphorus-containing derivatives exemplify how natural and semi-synthetic steroids can serve as promising leads for the development of next-generation anti-inflammatory and anticancer drugs. Future progress in this field will depend on integrated approaches combining biosynthetic studies, advanced spectroscopic and structural analysis, cheminformatics-based activity prediction, and detailed mechanistic pharmacology, including well-defined in vitro and in vivo models.

In conclusion, the convergence of anti-inflammatory and anticancer activities is a defining and exploitable feature of many structurally unique steroids. A deeper understanding of the chemical determinants and biological mechanisms governing this duality will be essential for the rational design of steroid-based therapeutics aimed at inflammation-driven malignancies and other complex, multifactorial diseases.

## 14. Future Perspective

From a translational and clinical standpoint, steroidal compounds with dual anti-inflammatory and anticancer activity offer a particularly attractive therapeutic strategy, as they may simultaneously suppress tumor-promoting inflammation while directly inhibiting malignant cell growth. Such dual-action agents could reduce reliance on combination therapies, lower cumulative toxicity, and improve patient outcomes in inflammation-associated cancers, including colorectal, liver, pancreatic, breast, and prostate malignancies. Several structural classes discussed in this review—most notably withanolides, halogenated marine steroids, epoxy- and peroxy-steroids, and phosphorylated derivatives—display pharmacological profiles compatible with further preclinical development. Future efforts should prioritize rigorous in vivo validation, pharmacokinetic and toxicity profiling, and the identification of predictive biomarkers for patient stratification. In addition, advances in steroid formulation, targeted delivery, and structure-guided optimization may facilitate the clinical translation of these compounds. Ultimately, integrating natural-product-inspired steroid chemistry with modern medicinal chemistry and systems pharmacology holds significant promise for the development of next-generation therapeutics addressing both cancer and chronic inflammatory disease.

## Data Availability

No new data were created or analyzed in this study.
